# Induced folding in RNA recognition by *Arabidopsis thaliana* DCL1

**DOI:** 10.1093/nar/gkv627

**Published:** 2015-06-22

**Authors:** Irina P. Suarez, Paula Burdisso, Matthieu P. M. H. Benoit, Jèrôme Boisbouvier, Rodolfo M. Rasia

**Affiliations:** 1 Instituto de Biología Molecular y Celular de Rosario. 27 de Febrero 210 bis, predio CCT, 2000 Rosario, Argentina; 2 Área Biofísica, Facultad de Ciencias Bioquímicas y Farmacéuticas, Universidad Nacional de Rosario. Suipacha 531, 2000 Rosario, Argentina; 3 CEA, Institut de Biologie Structurale Jean-Pierre Ebel, Grenoble, France; 4 CNRS, Institut de Biologie Structurale Jean-Pierre Ebel, Grenoble, France; 5 Université Joseph Fourier – Grenoble 1, Institut de Biologie Structurale Jean-Pierre Ebel, Grenoble, France

## Abstract

DCL1 is the ribonuclease that carries out miRNA biogenesis in plants. The enzyme has two tandem double stranded RNA binding domains (dsRBDs) in its C-terminus. Here we show that the first of these domains binds precursor RNA fragments when isolated and cooperates with the second domain in the recognition of substrate RNA. Remarkably, despite showing RNA binding activity, this domain is intrinsically disordered. We found that it acquires a folded conformation when bound to its substrate, being the first report of a complete dsRBD folding upon binding. The free unfolded form shows tendency to adopt folded conformations, and goes through an unfolded bound state prior to the folding event. The significance of these results is discussed by comparison with the behavior of other dsRBDs.

## INTRODUCTION

Small RNA molecules have emerged as a major mechanism of gene regulation in higher eukaryotes. MicroRNAs are 21 nucleotides molecules processed from endogenous transcripts that perform their function within an effector RNA-induced silencing complex (RISC), in which their role is to recognize target mRNAs through base pair complementarity. Recognition of mRNAs by the RISC gives rise to translational repression or to degradation of the corresponding transcript (1).

MiRNAs are excised from longer precursors (pri-miRNAs), which are transcribed in the nucleus by RNA polymerase II. The actual miRNAs are located within stem-loop structures in the pri-miRNA and are released through the action of RNase III-type enzymes. In plants the enzyme DICER-LIKE1 (DCL1) excises the mature miRNA in at least two steps, aided by the dsRNA-binding protein HYL1 and the zinc-finger protein SERRATE (2–4), forming the microRNA processing complex. Although these global aspects of miRNA processing are established, there is little information on the detailed structural features underlying the formation of the processing complex and the recognition of the pri-miRNA substrates.

The proteins that form the microRNA processing complex in plants are modular, containing several domains that could participate in substrate binding. Among them there are four double stranded RNA binding domains (dsRBDs), two in HYL1 and two in DCL1. These domains are characterized by an elongated fold, with a topology α-β-β-β-α, where the two alpha helices are packed on the same side of the antiparallel beta sheet (5,6). DsRBDs interact with dsRNA segments of ca. 12–16 bp, corresponding to 1.5 turns of dsRNA helix. Binding of dsRBDs to RNA is mediated by three regions of the protein: a conserved sequence in helix 1, the loop between strands 1 and 2 and the N-terminus of helix 2. The residues in these regions that interact with the RNA bind primarily to the phosphate backbone and to 2′ OH groups of the ribose moieties (5,6). In this way, binding of dsRBDs is in principle not sequence-specific. However, it was recently shown that some dsRBDs can actually recognize particular features of the RNA structure, like mismatched bases or loops (7–9). DsRBDs are usually found in tandem, giving rise to an enhancement in affinity or specificity, or alternatively, allowing for functional divergence of the motifs resulting in some dsRBDs specializing in protein–protein interactions (5,6). In DCL1 the two dsRBDs are located in tandem at the C-terminus and were shown to be essential for the function of the protein in pri-miRNA processing. Truncation of the sequence at the level of the second domain (*dcl1–9* mutant) strongly diminishes miRNA processing, resulting in plants with a clear phenotype. In *dcl1–9* mutant flowers, the central region of the floral meristem remains in an indeterminated state. Plants produce defective ovules and the mutation affects most plant organs morphologically (10). Furthermore, an insertion mutant that truncates the protein at the first domain (*dcl1–6* mutant) is embryonic lethal, presumably because it abolishes DCL1 activity (11). This suggests that the first dsRBD (DCL1-A hereafter) is crucial for the activity of DCL1 in the context of the miRNA-processing complex. More recently, a construct containing the two tandem dsRBDs of DCL1 was shown to complement *hyl1–2* mutant plants, further demonstrating the importance of this region in substrate recognition and in the formation of the miRNA processing complex in plants (12). In a previous work we characterized the second dsRBD of DCL1 from *Arabidopsis thaliana* (DCL1-B hereafter) (13). Here we focus on the functional and structural features of the first dsRBD of the same protein.

## MATERIALS AND METHODS

### Protein expression and purification

DNA coding for DCL1-A and DCL1-AB were amplified by polymerase chain reaction from a cDNA library. A synthetic gene optimized for expression in *E. coli* was purchased for mouse Dicer dsRBD (GenScript, USA). All three genes were cloned into the pET-TEV expression vector (14). Site-directed mutations were obtained by using the whole plasmid amplification method (15); the primers used are shown in the Supplementary Information. All mutations were confirmed by DNA sequencing. The plasmids were transformed in *E. coli* BL21 (DE3) cells, which were then grown at 37°C in M9 minimal medium supplemented with either 1 g/L ^15^NH_4_Cl or 1 g/L ^15^NH_4_Cl and 2 g/L [U-^13^C]glucose (Cambridge Isotope Laboratories). For perdeuterated samples, cultures were grown on 100% D_2_O M9 minimal medium supplemented with 1 g/L ^15^NH_4_Cl and 2 g/L [U-^2^H-^13^C]glucose. Protein expression was induced at OD_600_ ≈ 0.7 and cells were grown overnight at 25°C. The cells were harvested by centrifugation. Cell pellets were resuspended in 50 mM Tris (pH 8.0), 500 mM NaCl, 5 mM imidazole, and 1 mM β-mercaptoethanol and lysed by sonication. The clarified supernatant was purified using a Ni(II)-affinity column and the protein was eluted with the same buffer supplemented with 350 mM imidazole. Fractions containing the protein were concentrated and digested with His-tagged TEV protease (14). The protease was removed with a Ni(II)-affinity column, and the protein was further purified by a G75 size exclusion chromatography column equilibrated with 100 mM Phosphate, 50 mM NaCl, 10 mM β-mercaptoethanol, pH 7.0, in the case of DCL1-A and mouse Dicer, or by an ion exchange chromatography step on a CM-sephadex column, for DCL1-AB. All purification steps were followed by SDS-PAGE. Before being used, protein samples were exchanged into the appropriate buffer employing PD10 desalting columns according to manufacturer's instructions (GE Healthcare). Protein concentration was measured by UV absorption at 280 nm using the corresponding absorptivity coefficient calculated by ProtParam tool at ExPASy web portal (16).

### RNA samples production

RNA samples were produced by *in vitro* transcription with T7 RNA polymerase, using annealed oligonucleotides for short constructs and linearized plasmid for long constructs. Briefly, a mix was prepared containing 1X transcription buffer [40 mM Tris (pH 8), 5 mM DTT, 1 mM spermidine, 0.01% Triton X-100 and 80 mg/ml PEG 8000], each rNTP at 4 mM (rA, rC, rG and rU), 20 mM MgCl_2_, 40 μg/ml BSA, 1 unit of pyrophosphatase and the annealed template at 35 μg/ml. The reaction was started by addition of T7 RNA polymerase and allowed to proceed for 3 h at 37°C. Then, 50 units of RNase-free DNase were added, and the mix was incubated further for 30 min at 37°C. The reaction mixture was then diluted 8-fold in 20 mM Tris, 10 mM EDTA, and 8 M urea (pH 8.0) and loaded on a Q-Sepharose column equilibrated with the same buffer. The column was eluted with a gradient from 0 to 1 M NaCl in the same buffer. Fractions containing RNA, as determined by A_260_, were checked via denaturing 5% polyacrylamide gel electrophoresis. The fractions with the desired transcript were pooled, dialyzed three times against 200 volumes of H2O, and lyophilized for storage. Before being used, the RNA samples were dissolved in the appropriate buffer and annealed by being heated to 100°C and flash-cooled in an ice/water bath. RNA and DNA samples concentrations were estimated by measuring absorption at 260 nm in 8M urea, to avoid errors induced by hypochromic effect, and using the extinction coefficient calculated by the OligoCalc webserver (17).

### Fluorescence anisotropy assays

**Figure 1.**
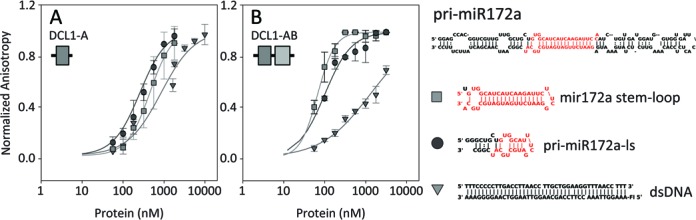
RNA and DNA binding by DCL1-A (**A**) and DCL1-AB (**B**) followed by fluorescence anisotropy. The sequence and secondary structure of pri-miR172a and of the constructs used are shown on the right.

RNA fragments were labeled with fluorescein using the 5′ EndTag Nucleic Acid Labeling System and fluorescein maleimide thiol reactive label from Vector Laboratories. Fluorescein labeled DNA was obtained from Sigma. Fluorescence anisotropy was measured on a Varian Cary Eclipse spectrofluorometer exciting the sample at 492 nm and measuring emission at 520 nm. Anisotropy values were obtained from the average of three measurements with an integration time of 20 s. Titrations were performed in 10 mM phosphate pH 7.0, using a 50 nM solution of labeled nucleic acid to which the protein was added. Each set of experimental data points shown in Figure 1 was obtained by calculating the average value of three independent measurements. Error bars show the standard error of the measurement. Experimental data points (r) were fitted to a hyperbolic function, using a single-site binding model:

\begin{equation*} {\rm r} = {\rm r}_0 + {\rm a}*[{\rm P}]/({\rm b} + [{\rm P}]) \end{equation*}

where ‘[P]’ corresponds to free protein concentration, ‘r_0_’ is the anisotropy of the free RNA (or DNA), ‘a’ is the amplitude of the change in anisotropy upon binding and ‘b’ is the apparent dissociation constant. Titration curves were normalized for plotting by subtracting from each data point the value of ‘r_0_’ and dividing the result by the amplitude ‘a’.

### NMR experiments

Unfolded free DCL1-A backbone resonance assignment: backbone ^1^H, ^13^C and ^15^N chemical shifts of free DCL1-A were assigned using a set of triple-resonance spectra (BEST-HNCA/(CO)CA, BEST-HNCACB/(CO)CACB, BEST-HN(CA)CO/HNCO, HN(CA)HA) (18) collected on a 600 MHz Bruker spectrometer. All spectra were processed with NMRPipe (19) and analyzed with CCPNMR (20). Chemical shifts were referenced with respect to the H_2_O signal at 4.77 ppm (pH 6.5, 25°C), using the H:X frequency ratios of the zero point according to Markley *et al*. (21). The assignments were deposited in the BioMagResBank (BMRB ID: 19105). Buffer conditions were 10 mM phosphate buffer (pH 6.5), 1 mM β-mercaptoethanol, 10 mM EDTA, 1X cOmplete (protease inhibitor cocktail, Roche) and 10% D_2_O. ^1^H-^15^N heteronuclear NOE data sets were acquired at 600 MHz using the standard Bruker pulse sequence.

Folded DCL1-A resonance assignment and structure calculation: backbone ^1^H, ^13^C and ^15^N chemical shifts were assigned using a set of standard triple-resonance spectra collected on a 600 MHz Varian spectrometer equipped with a cryogenically cooled probe. The sample was 400 μM perdeuterated, ^15^N-^13^C-labeled protein and 500 μM RNA in 20 mM phosphate buffer (pH 7.0) with 5 mM TCEP. The assignments were deposited in the BioMagResBank (BMRB ID: 19104). The fold of the bound protein was calculated using CS-Rosetta (22), following the protocol provided by the authors, using the HN, N, CA, CB and C’ chemical shifts as restraints.

ZZ Exchange Spectroscopy (ZZ-EXSY): ZZ exchange spectra were acquired on the perdeuterated DCL1-A sample in the presence of 1.25 equivalents of pri-miR172-ls (described above). Spectra were acquired with no mixing time and 0.2 s mixing time at 298 K using a Varian (Agilent) DirectDrive 800 MHz spectrometer.

Titration followed by NMR: spectra were acquired at 298 K on a 600 MHz Bruker NMR spectrometer. Titrations were conducted in 10 mM phosphate (pH 6.5), 1 mM β-mercaptoethanol and 10% D_2_O. Aliquots of a 400 μM pri-miR172-ls solution were added to a 200 μM ^15^N-labeled DCL1-A sample. At each step, a ^1^H 1D spectrum, including the low field imino proton region, and a ^1^H−^15^N SOFAST-HMQC (23) spectrum were acquired.

RNA Imino ^1^H assignment: natural abundance 1H-15N HSQC and 1H-1H NOESY (mixing time 150 ms) spectra of pri-miR172-ls RNA in 20 mM Cacodylic acid buffer pH 6 were acquired. The signals were assigned by means of a NOESY walk (Supplementary Figure S10).

### Circular Dichroism Spectroscopy

CD spectra were acquired on a Jasco J-810 spectropolarimeter. The samples were placed on a 0.1 cm path cuvette to minimize the buffer contribution to absorption. Spectra were acquired from 200 to 300 nm, averaging eight scans to improve the signal to noise ratio. Buffer condition was 10 mM phosphate (pH 7). Protein concentration was 8 μM and RNA was added in 0.25 equivalent steps for protein titrations with pri-miR172a-ls. For pri-miR172a titration with DCL1-A, aliquots of a 45 uM solution of DCL1-A were sequentially added to a 1 uM sample of the full-length precursor. The complete sets of titration spectra were analyzed by multivariate curve resolution–alternating least-squares (MCR-ALS) (24) in order to obtain the spectra of the components that develop in the mixture.

### Structural alignment

The structural alignment was performed using The PyMOL Molecular Graphics System, Version 0.99.

## RESULTS

### Both DCL1 dsRBDs participate in the recognition of substrate RNA

In order to understand the participation of the first dsRBD of DCL1 (UniProt ID: Q9SP32) in substrate binding and recognition we produced protein constructs corresponding to this region of the protein (DCL1-A, residues M1732-N1811), and to the double domain (DCL1-AB, residues M1732-S1909). We then determined the binding affinities of both constructs for substrate RNA by means of fluorescence polarization assays (Figure 1 and Table 1). We tested two different regions of the miR172a precursor as binding partners, the lower stem region (pri-miR172a-ls), that was previously shown to be essential for the correct processing of miRNA precursors (25), and the miRNA/miRNA* region (miR172a stem-loop).The affinity of the isolated DCL1-A construct for pri-miR172a fragments is similar to the one determined previously for DCL1-B (13). DCL1-A RNA binding affinity is somewhat higher for the pri-miR172a-ls construct than for the miR172a stem loop. The secondary structure of the former is less regular than that of the latter, including one mismatch and three G•U wobble base pairs, thus suggesting that DCL1-A may have some preference for imperfect dsRNA segments. In contrast, the double domain DCL1-AB shows an affinity one order of magnitude higher for the miR172a stem-loop and threefold higher for the primiR172a-ls construct, indicating that both domains participate jointly in the recognition of the substrate.

**Table 1.** Nucleic acid binding affinities of DCL1 dsRBD constructs obtained by fitting experimental data points from fluorescence anisotropy assays

	pri-miR172a-ls	miR172a	dsDNA
DCL1-A	300±50 nM	723±250 nM	860±270 nM
DCL1-B^a^	350±20 nM	810±180 nM	600±50 nM
DCL1-AB	100±11 nM	67±9 nM	680±110 nM

^a^From reference (13).

We have previously established that DCL1-B binds dsDNA, which is unusual for dsRBDs (13). Prompted by those results, we tested DCL1-A and DCL1-AB for dsDNA binding as well. We found that both constructs do bind dsDNA, showing that DCL1-A is also peculiar in terms of nucleic acid recognition with respect to other dsRBDs. The affinity of the double domain for dsDNA is similar to that for both isolated domains, indicating that in this case the domains behave as independent binding units. This contrasts with the situation found for substrate RNA, therefore showing that the presence of two tandem dsRBDs in the C-terminus of DCL1 could contribute to the discrimination of substrate RNA against other types of nucleic acids.

### DCL1-A is intrinsically disordered

**Figure 2.**
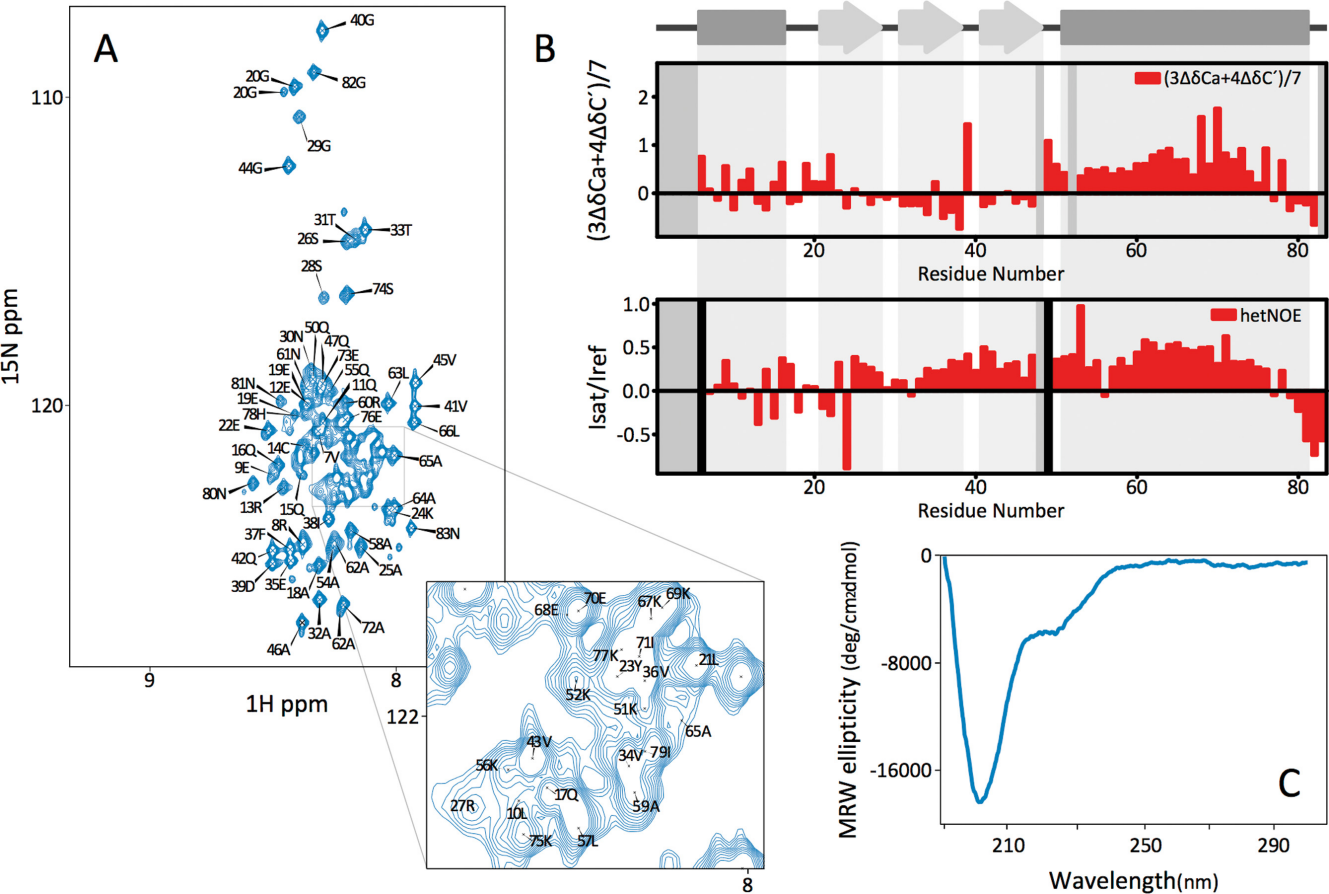
Spectroscopic characterization of the free unfolded form of DCL1-A. (**A**) ^1^H-^15^N-HMQC spectrum. (**B**) Combined CA and C’ secondary chemical shifts (top) and ^1^H-^15^N heteronuclear NOEs (bottom). Secondary structure elements of the folded form are schematized on top. Vertical black bars indicate Pro residues and gray bars indicate unassigned resonances. (**C**) UV-CD spectrum.

We then decided to study the system from a structural point of view, to gain insight into the recognition mode of the protein. Quite unexpectedly, the CD spectrum of DCL1-A indicates that the polypeptide is disordered (Figure 2C). We expressed the protein construct labeled with ^15^N, and we found that the ^1^H-^15^N-HMQC spectrum of DCL1-A shows a very limited dispersion, confirming that this domain does not fold by itself (Figure 2A). This result contrasts with the disorder prediction of DCL1 dsRBDs, which indicates that both domains should be folded to a similar extent (MetaPrDOS (26), Supplementary Figure S1). To explore the possibility that the domain boundaries may not have been well defined, we obtained a labeled sample of DCL1-AB and we produced another construct extending DCL1-A to the N-terminal side, including 25 residues from the preceding RNAseIII-2 domain (DCL1-A/N, residues G1707-G1810). In the ^1^H-^15^N-HMQC spectra of the constructs, neither the signals corresponding to DCL1-A nor those arising from DCL1-B were affected. This demonstrates that both DCL1 dsRBDs are independent from each other, and that the presence of flanking regions of DCL1 is not enough to allow DCL1-A to acquire a folded conformation (Supplementary Figure S2).

Having established that DCL1-A is disordered in solution we resorted to NMR observables to obtain information at the residue level on the structural propensities of different regions of the protein. It is well known that several intrinsically disordered proteins (IDPs) show some degree of dynamic preorganization that lead to the preference for conformations which yield preformed binding sites (27). We first obtained ^1^H-^15^N-HMQC spectra at different temperatures from 298 to 278 K with 5 K intervals, and found that the dispersion remains poor, showing that the protein is unfolded even at low temperatures (Supplementary Figure S3). Despite showing a poorly dispersed ^1^H-^15^N-HMQC spectrum DCL1-A appears to have some residual structure, since titrating the protein with Urea results in substantial chemical shift changes for most of the signals. The final state (8M Urea) shows a much better defined spectrum (Supplementary Figure S3), characteristic of fully unfolded proteins, with narrow lines due to fast conformational averaging. This suggests that the free protein is not completely unstructured.

We then obtained the resonance assignment of the protein backbone atoms using standard triple resonance experiments. The deviation of the chemical shifts of CA and C’ atoms from their typical random coil values are good indicators of the presence of residual secondary structure elements in the protein. We found that the N-terminal half of the protein shows no or little tendency to populate folded conformations, but the C-terminal half displays some tendency to populate strand or helical structures (Figure 2B). The secondary structure propensity is also coincident with the regions predicted to correspond to alpha helix 2 and beta strands 2 and 3 in the dsRBD fold based on sequence alignment of DCL1-A with dsRBDs of known structure (Supplementary Figure S4). Overall, these results suggest that the free protein populates transiently folded conformations that correspond in part to the expected secondary structure of a dsRBD.

^1^H-^15^N NOEs are sensitive to the local correlation time of the amide groups, thus reporting on the rigidity of the polypeptide backbone. The data for DCL1-A show that the protein is overall flexible presenting low positive values of NOE, but the N-terminal region seems to be less structured than the C-terminal half (Figure 2B). This result is in agreement with the observation of secondary structure propensity in the region corresponding to beta strands 2 and 3 and alpha helix 2.

Among dsRBDs for which 3D structures are available the closest homologs of DCL1-A are the dsRBDs of mouse Dicer (PDB ID: 3C4B) and of *Kluyveromyces polysporus* Dicer (PDB ID: 3RV0) (Supplementary Figure S4) (28,29). It is noteworthy that, despite the relatively high sequence homology with the intrinsically disordered DCL1-A (53% homology with mouse Dicer and 35% homology with *K. polysporus* Dicer), both dsRBDs show well-defined structures in the crystal. The dsRBD in *K. polysporus* Dicer is connected by a flexible linker to the rest of the protein and is separated from the RNAseIII domain within the asymmetric unit in the crystal, indicating that it is an independent folding unit. However in the crystal structure of mouse Dicer the dsRBD and the neighboring RNAseIII domain are close and share some buried surface, which could help to stabilize its structure. In order to confirm whether the isolated mouse Dicer dsRBD is structured, we expressed the protein and acquired a ^1^H 1D NMR spectrum. The spectrum shows well-dispersed amide signals and upfield-shifted signals corresponding to methyl groups in folded regions, indicating that mouse Dicer dsRBD is an independent folding unit (Supplementary Figure S5). The dsRBD of *Schizosaccharomyces pombe* Dicer was recently shown to be stably folded as well (30). Therefore the intrinsic disorder in the free form seems to be a feature exclusive to DCL1-A among the structurally characterized Dicer dsRBDs.

### DCL1-A folds in the presence of substrate

Despite being mostly unstructured, DCL1-A does bind to RNA and DNA, and cooperates with DCL1-B within the double domain. We therefore asked ourselves whether binding to the target would require the protein to adopt a folded conformation.

**Figure 3.**
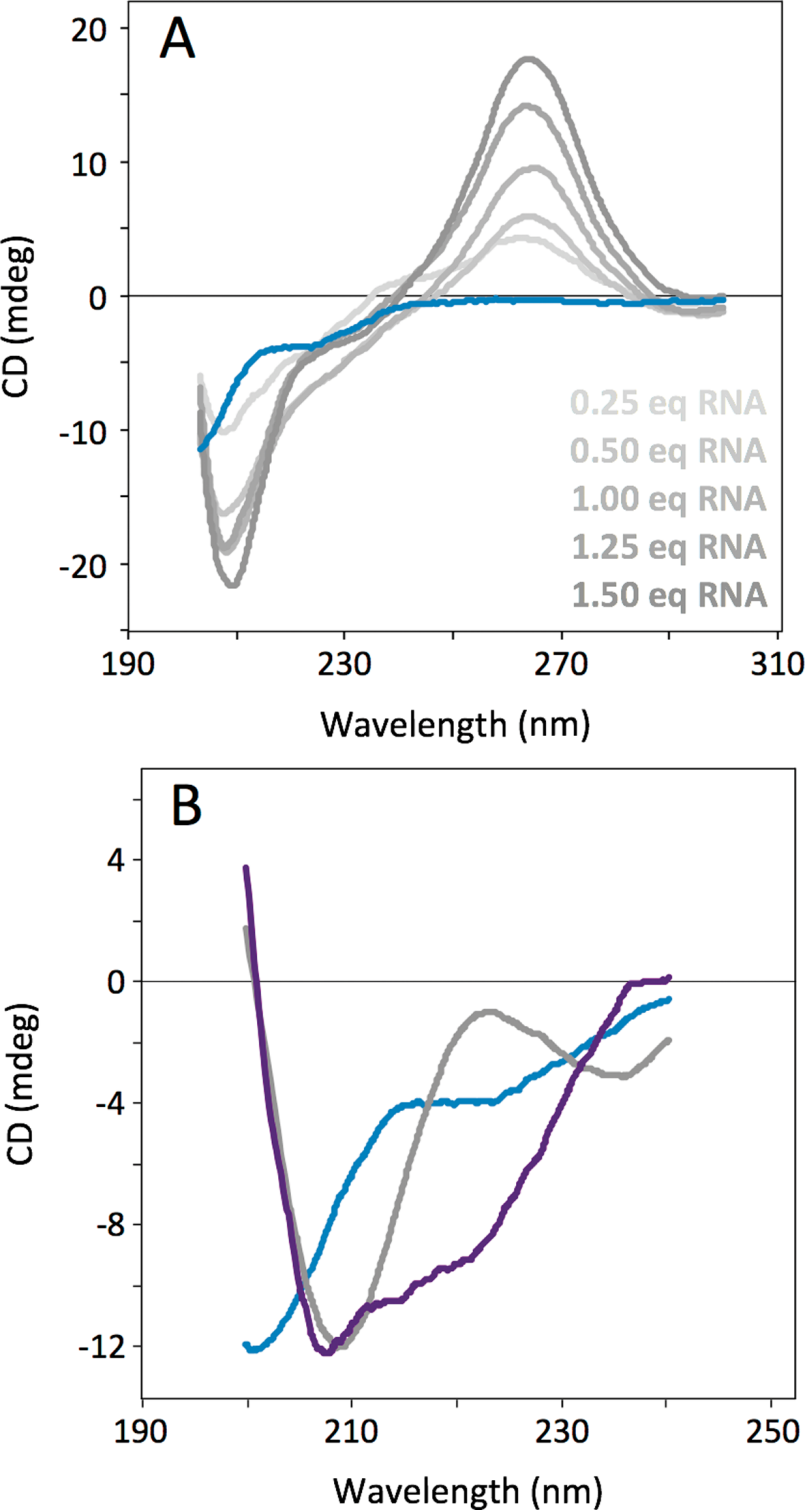
Binding of DCL1-A to RNA followed by CD spectroscopy. (**A**) Analysis of secondary structural changes by CD. The blue spectrum corresponds to the free protein. Increasing gray spectra correspond to increasing amounts of added dsRNA (0.25 equivalents RNA:Protein on each step). (**B**) Calculated CD spectra of the components that develop in the mixture obtained by MCR-ALS. Blue, unfolded DCL1-A, gray, RNA, purple, folded DCL1-A.

We first tested this by following the formation of DCL1-A:RNA complex by CD spectroscopy (Figure 3A) using the pri-miR172a-ls construct. The free protein shows the characteristic spectrum of unfolded polypeptides, with a negative minimum at ca. 200 nm. Addition of RNA immediately changes the spectrum in the far UV region, but due to the intense negative band of dsRNA at 210 nm it is hard to determine if the protein develops secondary structure. Therefore we analyzed the spectra in the titration by multivariate curve resolution–alternating least-squares (MCR-ALS) (24), and found three components in the titration, indicating that the formation of the complex leads to a different species (Figure 3B). Two of the basis spectra correspond well with the spectra of the free protein and of the free RNA, whereas the third component closely matches the spectrum of canonical dsRBDs (Supplementary Figure S6), suggesting that DCL1-A acquires the same secondary structure as other stably folded homologs. We then monitored binding of DCL1-A to full-length pri-miR172a using the same methodology. We titrated up to 10 equivalents DCL1-A on the RNA sample. In this case there is a larger contribution arising from the RNA component in the spectra, because of the larger size of the RNA construct. However, the contribution of the folded protein component to the spectra can clearly be noticed at 10 equivalents added protein. Analysis of the spectra by MCR-ALS shows that the folded protein component increases up to the higher protein:RNA ratio measured (10:1), indicating that the longer precursor can harbor several equivalents of DCL1-A and suggesting that the protein has no preference for any particular region of the precursor (Supplementary Figure S7). We used the same method to study the ionic strength dependence of dsRNA binding by DCL1-A. We found that the complex dissociates at NaCl concentrations above 50 mM (Supplementary Figure S7), showing the appearance of the spectrum of the free unfolded protein, demonstrating that formation of the complex has a large electrostatic component, as observed for DCL1-B as well (13).

**Figure 4.**
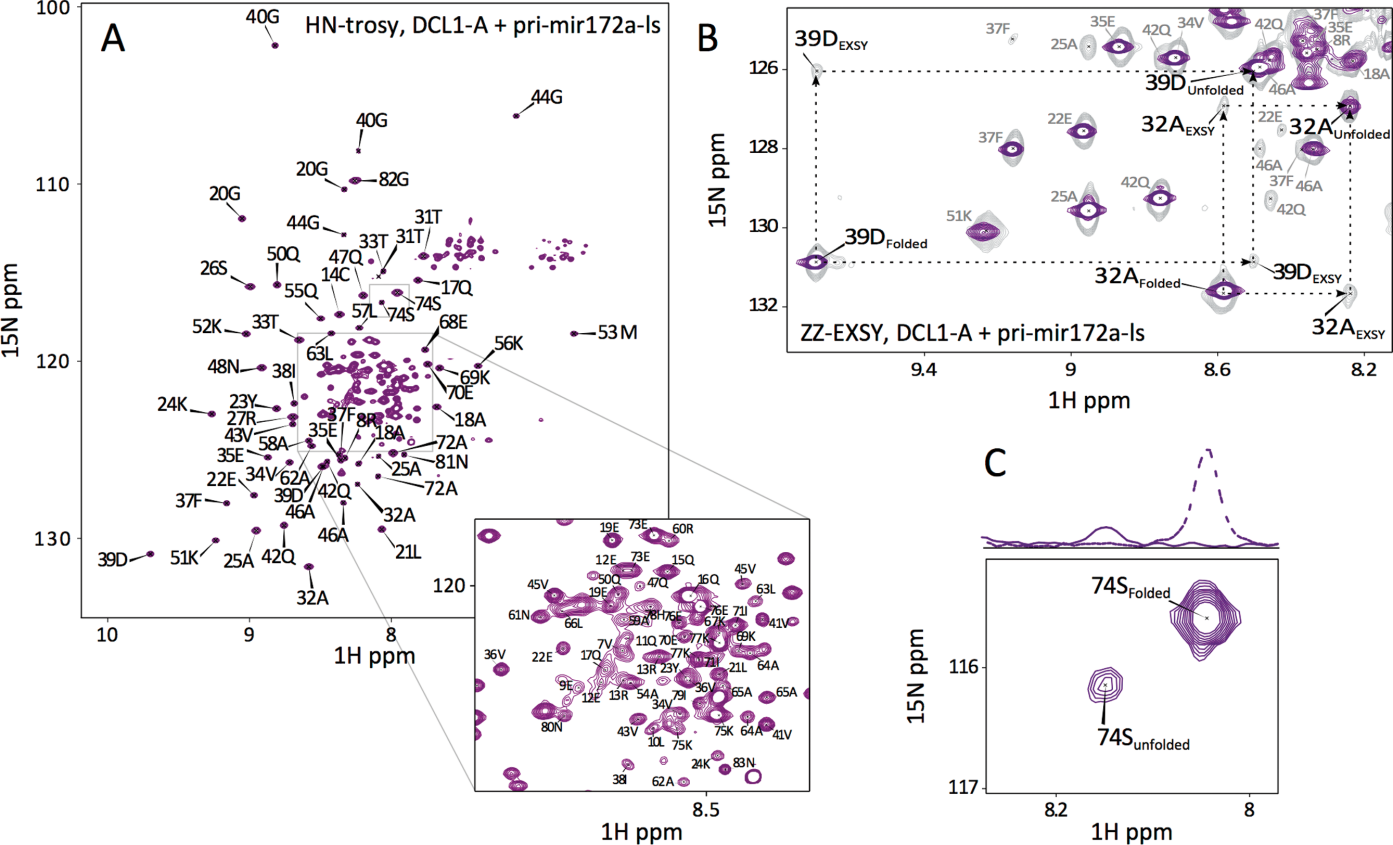
NMR characterization of the folded form of DCL1-A. (**A**) ^1^H-^15^N-Trosy spectrum of the complex between DCL1-A and 1.25 equivalents of pri-miR172a-ls. (**B**) Detail of the ZZ exchange spectrum (0.2 s mixing time, gray) superimposed with ^1^H-^15^N-Trosy spectrum (purple), dashed lines depict rectangular pattern between signals of unfolded and folded forms of DCL1-A. (**C**) Spectrum detail depicting the ratio of 5 between the signal of unfolded and folded forms of the protein.

In order to better characterize the folded form of DCL1-A, we proceeded to study the protein in complex with 1.25 equivalents of pri-miR172a-ls by NMR. To avoid line broadening due to the relatively large size of the complex (ca. 21 KDa) we resorted to a perdeuterated ^15^N-^13^C-labeled protein sample. The ^1^H^15^N-TROSY spectrum of the complex displays a new set of well-dispersed signals, showing that the protein acquires a more ordered fold in the bound form (Figure 4A). The new set of signals coexists with the signals corresponding to the unfolded protein, showing that the free and bound forms of the protein are in slow exchange in the NMR timescale. In order to confirm that both protein species are functional and in conformational exchange between each other we acquired ZZ exchange spectra on the sample and found the corresponding exchange crosspeaks, indicating that the folded and unfolded form are in equilibrium (Figure 4B and Supplementary Figure S8).

The presence of a single set of dispersed signals indicates that DCL1-A forms a well-defined complex with substrate RNA. This contrasts with the complex formed by DCL1-B and by mouse Dicer dsRBD, where the signals from the protein disappear upon binding to RNA, indicating either the formation of heterogeneous complexes or the presence of intermediate exchange (13,31).

We decided to verify if the substrate-induced folding of DCL1-A was specific of dsRNA or it could be simply due to the effective increase of the ionic strength of the solution caused by the addition of the polyanion. Increasing the NaCl concentration up to 500 mM does not lead to major changes in the 1H-15N HSQC spectrum of DCL1-A (Supplementary Figure S9). The same is observed in the far UV CD spectrum. Knowing that DCL1-A binds dsDNA we wondered if this other nucleic acid could induce DCL1-A folding. The CD spectra of DCL1-A:DNA complexes show a slight shift in the minimum from 200 to 202 nm, and a small variation in the CD_200nm_/CD_220nm_ ratio, demonstrating that binding to DNA induces a variation in the conformational sampling of the protein, but this still remains disordered (Supplementary Figure S9). These experiments indicate that DCL1-A folding is specifically induced by dsRNA.

**Figure 5.**
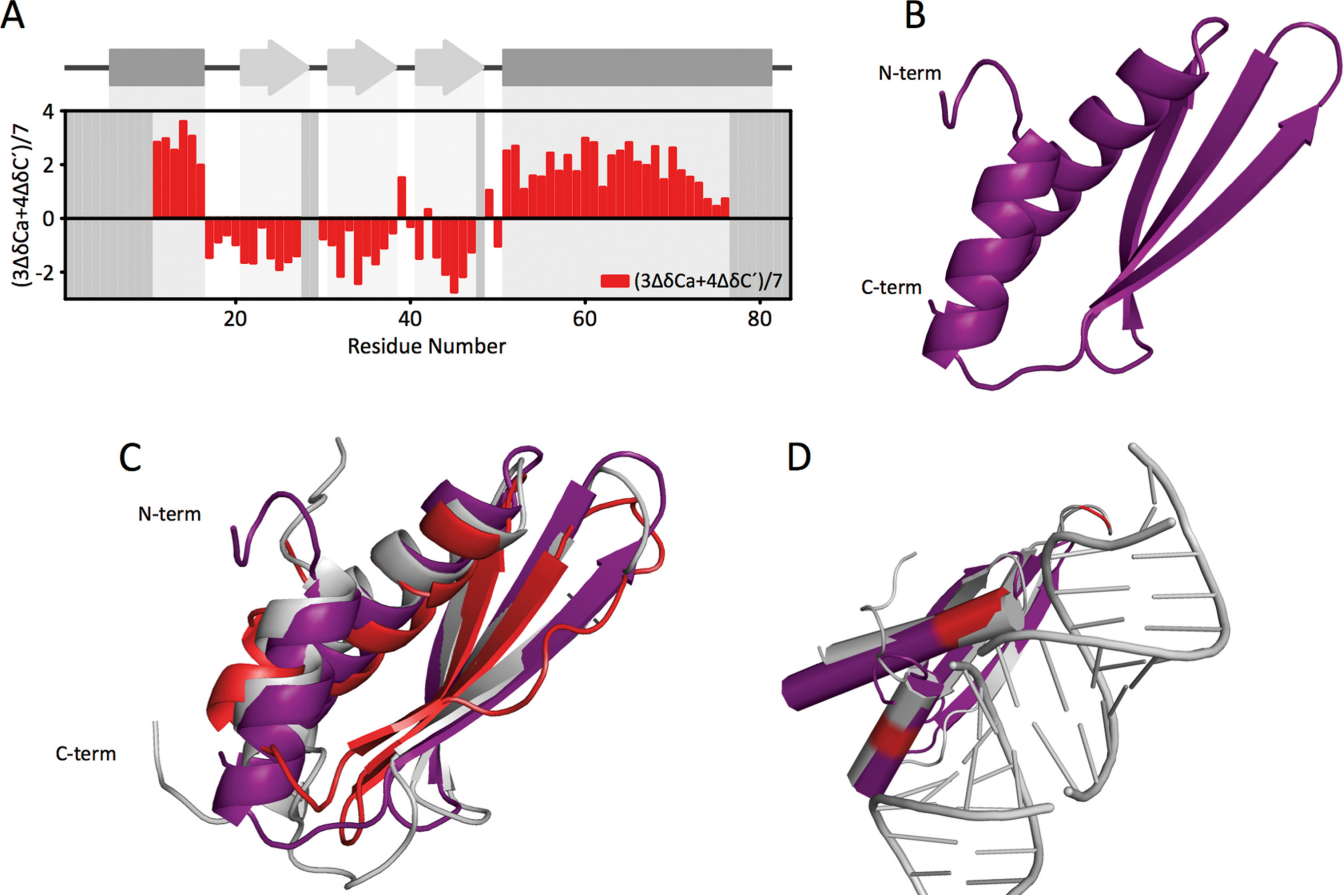
Calculated structure of the folded form of DCL1-A. (**A**) Combined secondary backbone chemical shifts of the folded form of DCL1-A in complex with 1.25 equivalents of pri-miR172a-ls. (**B**) Calculated fold of DCL1-A. (**C**) DCL1-A (purple) structurally aligned with *M. musculus* Dicer dsRBD (PDB ID: 3C4B, red) and *K. polysporus* Dicer dsRBD1 (PDB ID: 3RV0, gray). (**D**) Superposition of DCL1-A (purple) with the *H. sapiens* TRBP-dsRBD2:dsRNA complex (PDB ID: 3ADL, gray). Residues that participate in the interaction with RNA are highlighted in red.

We then acquired a set of standard triple resonance spectra that allowed us to assign the backbone resonances of the structured form of the protein. The analysis of the secondary chemical shifts shows the presence of defined secondary structure regions that correspond with the α-β-β-β-α topology of canonical dsRBDs (Figure 5A). By using the assigned backbone chemical shifts we calculated the fold of the structured form employing CS-Rosetta (22). The 10 lowest rescored energy models converged to a dsRBD fold with an intra-RMSD of 1.14 ± 0.38 Å (Figure 5B). Comparison of the fold obtained for DCL1-A with the structures of other dsRBDs, by structural alignment, shows that two of the three RNA binding regions are well conserved (Figure 5D). However, the loop β1-β2 that recognizes the minor groove of dsRNA, is much shorter than that found in other homologous domains. In this sense DCL1-A is similar to the dsRBDs of mouse Dicer and that of the non-canonical Dicer protein of *K. polysporus*, the closest homologs among the dsRBDs of known structure (Figure 5C). The shorter loop was suggested to weaken the affinity of *K. polysporus* Dicer dsRBD for dsRNA due to the loss of a RNA binding determinant (29), but it is clear from our data that this feature does not hinder RNA recognition by DCL1-A.

**Figure 6.**
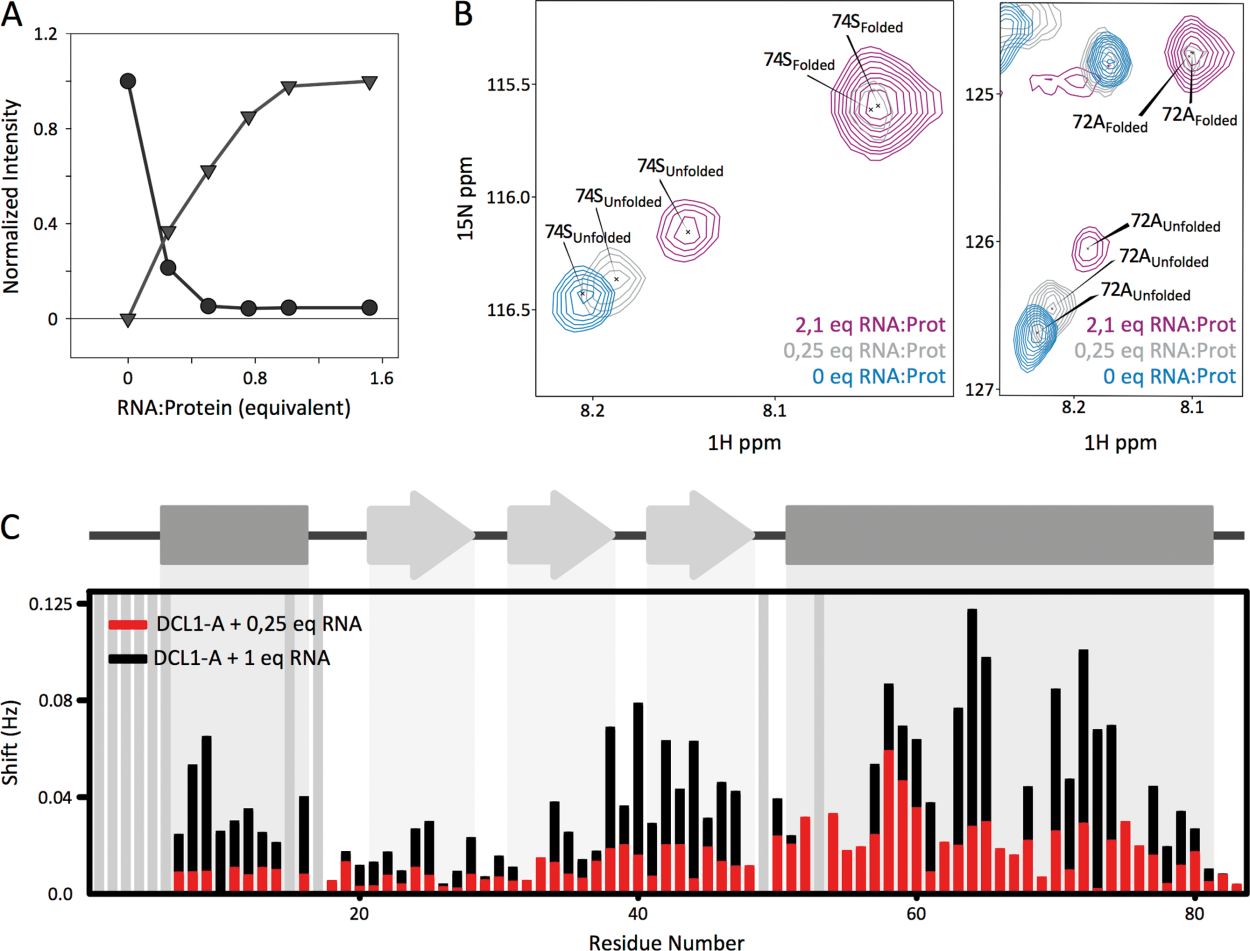
DCL1-A – RNA complex formation followed by NMR. (**A**) Evolution of the normalized averaged intensity of the signals in the ^1^H-^15^N-HMQC spectra during the titration corresponding to the unfolded (circles) and folded species (triangles). (**B**) Detail of the ^1^H-^15^N-HMQC spectra at different points of the titration depicting the shift of the unfolded form signals. (**C**) Chemical shift perturbation, calculated as ((Δδ-^15^N/6)2+(Δδ-^1^H)2)^1/2^, for each residue of the unfolded form in the presence of 0.25 equivalents (red) and 1 equivalent (black) of dsRNA with respect to the unfolded free form signal position.

In order to obtain a more detailed description of the complex formation we followed the titration of a ^15^N-labeled DCL1-A sample with pri-miR172a-ls dsRNA by NMR. For each titration point we acquired both a ^1^H 1D spectrum and a ^1^H-^15^N HMQC spectrum. The signals corresponding to the imino ^1^H of the RNA evolve slightly during the titration, differing from the spectrum of the free RNA (Supplementary Figure S10). Imino signals of the complex are broad, probably due to the larger correlation time with respect to the free RNA, and shifted in some cases, showing that binding of DCL1-A brings about small modifications in the RNA structure or dynamics. On the protein side, it is noteworthy that the signals corresponding to the unfolded form remain in the spectrum, even after addition of excess RNA (Figure 6A). The equilibrium constant between the folded and unfolded species was estimated to be ca. 5, based on the volume ratio of the signals corresponding to each species in the ^1^H-^15^N-HMQC spectrum of the perdeuterated sample (Figure 4C).

The presence of unfolded DCL1-A with excess RNA was rather unexpected, since a very minor fraction of the protein should be in its free form taking into account the dissociation constant obtained by fluorescence anisotropy titrations (300 nM). A closer analysis of the NMR titration experiment showed that the peaks corresponding to the unfolded form change their position upon addition of RNA (Figure 6B). The chemical shift changes observed for these peaks show no correlation with those brought about by an increase in salt concentration, ruling out a possible effect arising from the increase in ionic strength produced by the addition of the RNA polyanion (Supplementary Figure S9). This implies that a third protein species is formed, besides the free unstructured and the bound structured forms, which is in fast exchange with the unfolded free form. The narrow chemical shift dispersion of this species indicates that it is disordered as well. The linewidth of these signals is in average larger than that of the free form. These observations suggest that the third species corresponds to unfolded protein bound to the RNA. We followed the shifts of the signals during the titration in order to determine which regions of the unfolded protein were more affected by the interaction with the substrate. We found the largest changes on the regions corresponding to the N-terminal part of alpha helix 2 and beta strands 2 and 3 in the structured form (Figure 6C). These regions show some rigidity on the free unfolded polypeptide, as suggested by the heteronuclear NOE data. Analysis of the secondary chemical shifts of CA and C’ of this bound unstructured form shows that these regions acquire folded conformations to a greater extent than the free unstructured form (Supplementary Figure S11). All these observations suggest that the N-terminal region of helix 2 and beta strands 2 and 3 of the free protein are exploring the conformational space in such a way that allows DCL1-A to find its partner and form a complex which finally rearranges with the protein acquiring the folded conformation.

Based on these observations we propose the following model for the interaction of DCL1-A with substrate RNA:

\begin{equation*} {\rm F}_{\rm U} + {\rm R} \rightleftharpoons {\rm B}_{\rm U} - {\rm R} \rightleftharpoons {\rm B}_{\rm F} - {\rm R} \end{equation*}

where ‘F_U_’ is the Free Unfolded form, ‘R’ is the dsRNA, ‘B_U_-R’ is the Bound Unfolded form and ‘B_F_-R’ is the Bound Folded form.

**Figure 7.**
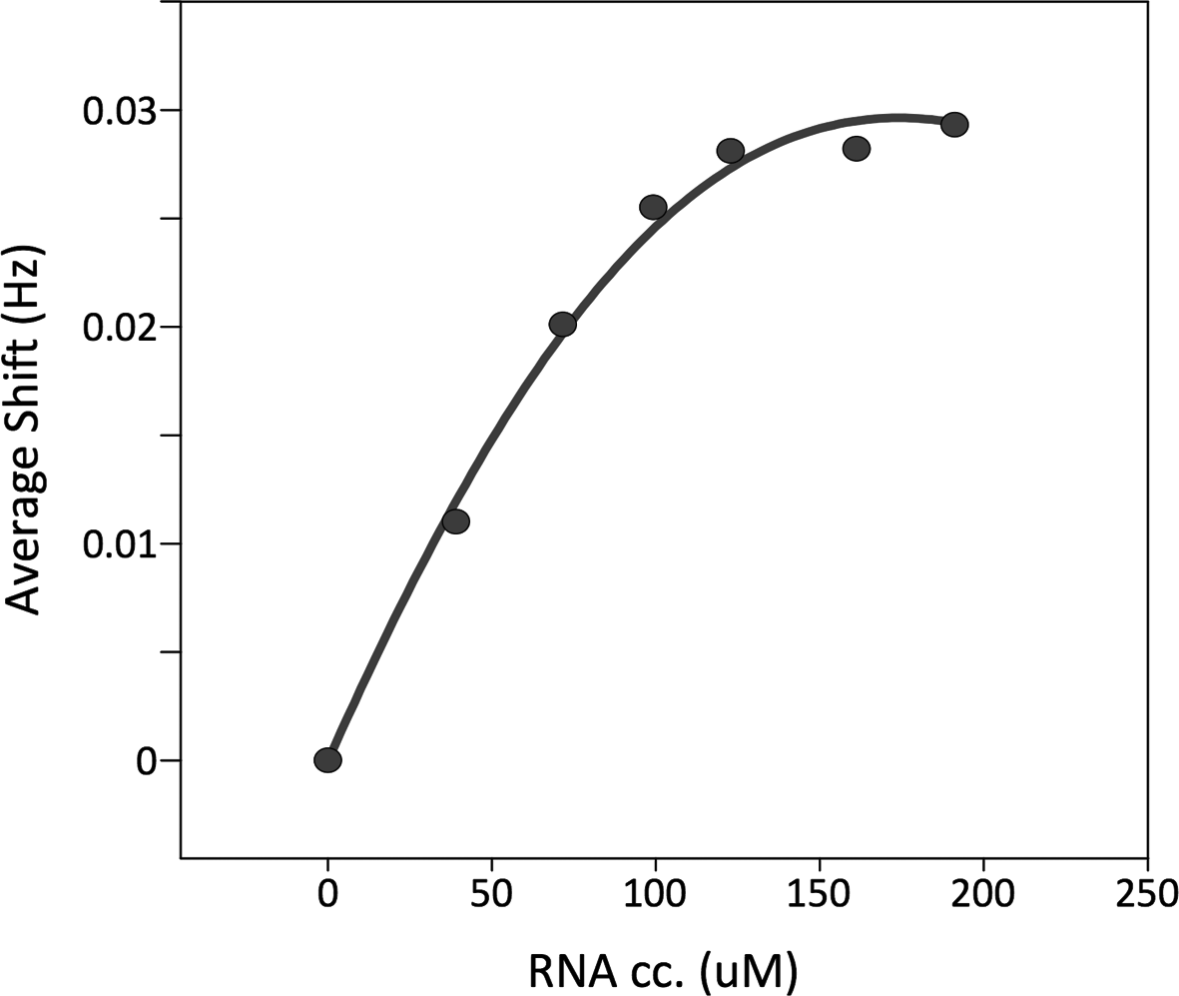
Average shift for all signals corresponding to the unfolded form of DCL1-A for each point of the titration (circles) and fitted curve considering a macroscopic dissociation constant of 300 nM and an equilibrium constant between the bound unfolded form and the bound folded form of ca. 5. The fitted microscopic dissociation constant for the complex formation of the unfolded form with the RNA was 2 uM.

By considering the values of the macroscopic dissociation constant obtained by fluorescence anisotropy (300 nM), where the signal reports on bound RNA, and the equilibrium constant between the bound unfolded and folded forms, estimated in ca. 5 based on the relative signal intensities of each species in the presence of excess RNA, the model predicts a value of 2 uM for the dissociation constant of the first step (see model in Supplementary Information). This low affinity constant would account for the fast exchange between the free and bound forms of the protein. We calculated the expected evolution of the concentration of ‘B_U_’ during the titration and fitted the data of the average shifts measured at each step for the whole protein. The calculated evolution shows an excellent agreement with the experimental data (Figure 7).

### On the low stability of the free folded form

Sequence analysis of DCL1-A by disorder prediction algorithms indicate that the domain should have a stable fold, but it is clear from our experimental results that the free protein is mostly disordered. These algorithms are broadly based on an analysis of the physicochemical characteristics of the protein sequence, meaning that the aminoacid order and composition of DCL1-A could very well result in a stably folded protein. The stability of a protein fold is determined by the contribution of a large number of both stabilizing and destabilizing forces, and the difference between them is usually relatively small with respect to the magnitude of the sum of individual interactions. Therefore the lower stability of free folded DCL1-A with respect to a free unfolded form could be due to the presence of a small excess of destabilizing contributions or the lack of necessary stabilizing contributions.

In order to understand the molecular basis of the low stability of the free folded form of DCL1-A, we performed a comparative analysis of DCL1-A with other dsRBDs of known structures. Sequence alignment of these proteins (Supplementary Figure S4) fails to highlight any particular residue or set of residues that could destabilize the fold of DCL1-A. Using the calculated fold for the bound form, we designed point mutations aiming at stabilizing the same fold in the free protein. We produced the following mutations: E1747W, as the presence of a bulky hydrophobic residue in this position was shown to stabilize the fold of a group of dsRBDs (32); Y1751F, in order to facilitate the incorporation of the aromatic sidechain into the hydrophobic core by eliminating the polar OH group; Q1783K, in the attempt to establish a pi-cation stabilization through the interaction with Y1751, as found in other dsRBDs; Q1778D to serve as N-terminal cap to helix alpha 2; and K1779A/K1780A, in order to neutralize the positive charged patch in the RNA binding region of DCL1-A. However we found that all of these mutants remain disordered (Supplementary Figure S12), indicating that the stabilization of the free folded form of DCL1-A probably requires a combination of all of these or more mutations.

## DISCUSSION

In the present work we have shown that the first dsRBD of DCL1 is quite uncommon among its homologs. The protein is intrinsically disordered, but it can still perform its expected role, binding dsRNA and working jointly with the second dsRBD in the recognition of the substrate.

The degree of structuring of proteins can be thought of as a continuum that ranges from stably folded proteins going through molten globules, locally structured proteins, and ending in fully disordered proteins (27,33–35). DCL1-A shows a CD spectrum featuring a clear minimum at 200 nm with some residual CD signal at 220 nm. The NMR spectra show poor dispersion, no medium range NOEs and relatively sharp signals, with secondary shifts showing partial helical population of in the C-terminus. These spectral features place DCL1-A as a locally structured disordered protein, without the stable secondary structure that characterizes molten globules (36–38) but more structured than largely disordered proteins (39–41).

Disordered proteins and disordered regions within proteins are currently known to have important roles in cellular processes where their inherent flexibility facilitates multiple target recognition or enhances the dynamics of complex formation (27,33,42,43). In many cases stretches of aminoacids, usually named molecular recognition elements, adopt regular structures when bound to other partners (44–50). Target recognition by disordered proteins can also lead to the acquisition of a more complex fold, although this effect is observed in fewer cases (51–53).

In terms of mechanism, the folding process of an IDP when bound to its partner can proceed via two pathways, induced fit or conformational selection (27). These mechanisms are limiting cases, most folding upon binding systems operating through an intermediate pathway where a partially preformed folded structure binds loosely to the partner and fits subsequently to the receptor site optimizing the interaction. The mechanism for DCL1-A binding and folding combines several characteristics described before for different systems. The unfolded free form transiently explores secondary structure elements that could be essential for its capability of binding to the substrate, as recently described for the activator for thyroid hormone and retinoid receptors or for the gamma-subunit of cGMP phosphodiesterase (54,55). The preexistence of residual secondary structure in DCL1-A may suggest that conformational selection could be operating in this case. However, the detection of an RNA bound species with chemical shifts denoting a disordered conformation indicates that the folding event appears to happen on the surface of the RNA being preceded by the formation of a lower affinity complex that is in fast exchange with the free protein. This is indicative of an induced fit mechanism operating in DCL1-A recognition of dsRNA (56). Similar results were obtained for other analogous systems where IDPs bind their substrates in a disordered encounter complex (48,49,57). In its final bound state DCL1-A acquires the canonical dsRBD fold, forming a well-defined complex with substrate RNA. Folded-bound and unfolded-bound proteins coexist in the presence of excess RNA, indicating that the difference in energy between both is low (ca. -4 kJ/mol when estimating the equilibrium constant as 5). Both bound protein forms are in slow exchange in the NMR timescale, suggesting the presence of a relatively large activation energy barrier between them. The relatively small energy difference between the unfolded bound and folded bound species, that accounts for the presence of measurable populations of both of them in equilibrium in the presence of excess RNA, suggests that the interaction with RNA is similar in terms of energy in both complexes. We believe that the presence of sizeable protein:RNA interactions in the unfolded bound species that have to be disrupted for the protein to acquire its folded conformation creates a large activation energy that causes the slow interconversion between the two forms.

Coupling of binding and folding has been demonstrated for many unstructured proteins, with several examples among RNA binding proteins (33,58–60). In many cases only short stretches of the IDPs fold, with fewer examples reported in which a protein acquires a fully folded state with a relatively complex topology upon partner recognition. Long protein extensions found in ribosomal proteins, that are disordered in the free state, are suggested to play a pivotal role in the assembly of the ribosomal particle (61). The protein NHP6A (51) is actually well-folded at 20°C, but loses structure at 37°C. It is similar to DCL1-A in the sense that it folds at 37°C in the presence of DNA, but the spectrum of the unfolded form at 37°C shows few signals suggesting that the protein behaves more like a molten globule under these conditions. In contrast, DCL1-A remains unfolded within the tested temperature range (from 5°C to 25°C). LEF-1, a member of the high-mobility group (HMG) family of proteins shows NMR spectra consistent with conformational heterogeneity, but forms a well-defined complex with its cognate DNA (53). In this case, CD spectra indicate that the free protein presents the same content of helical secondary as in the DNA-bound state, whereas the CD spectrum of free DCL1-A shows hardly any regular secondary structure content. To the best of our knowledge, this is the first case reported for a dsRBD folding upon dsRNA recognition.

What could be the purpose of DCL1-A being intrinsically disordered? DCL1-A folds upon binding to substrate RNA and its flexibility in the unbound state probably allows for the adaptation of the fold to the binding partner. Precursors of miRNA in plants are extremely variable in length, secondary structure and sequence (3,4). Bearing this in mind, we can speculate that the adaptability of DCL1-A could be crucial for the recognition of the very diverse set of substrates of DCL1. According to our model, the folding event is uncoupled from the low affinity binding event by a high activation energy barrier. This kind of mechanism has also been described before for other IDPs (62). Taking into account that DCL1 needs to search for its cleavage site within a large heterogeneity of precursors, we can propose that this low affinity binding step could allow the enzyme to explore different positions before tight binding to the substrate takes place through the folding step of DCL1-A.

Several IDPs can exert different functions depending on the cellular context or the molecular environment thanks to their highly dynamic conformation (62). It has been argued that coupled folding and binding can help the recruitment of the individual protein components during the formation of protein complexes. There are examples of dsRBDs whose function is not to bind dsRNA but rather to participate in protein–protein interactions (12,63–66). If DCL1-A was indeed necessary for the recognition of other protein partners, its marginal stability may have evolved to ensure the correct assembly of the miRNA-processing complex. On the other hand, intrinsically disordered proteins have been widely implicated in multiple target recognition, this function being facilitated by their capability of acquiring multiple conformations which allow for overlapping binding motifs and transient binding of different partners. Given this, the participation of DCL1-A in a function other than substrate RNA binding remains an intriguing possibility.

Intrinsically disordered regions are known to be essential to the functioning of scaffolding proteins, enhancing the plasticity of interaction by means of the fly-casting mechanism, easing in this way the formation of encounter complexes. While DCL1 is not itself a scaffolding protein, DCL1-B was shown to participate in intracellular localization of DCL1, as the truncated protein DCL1–9 fails to localize in dicing bodies (66). In this context, a possible function of the intrinsic disordered nature of DCL1-A could be to provide a long flexible linker between DCL1-B and the rest of the protein, facilitating in this way the recruitment of other partners within the nucleus. Once the complex is formed, DCL1-A could participate in substrate recognition by adopting its folded form in the presence of pri-miRNA.

Finally it is important to note that sequence alignment of plant DCLs shows that the sequence conservation within the first dsRBD from different plants is much higher in DCL1 than in DCL2 to 4, thus indicating that the particular features of DCL1 dsRBDs have originated early in evolution, as the sequence is highly conserved even in the moss *Physcomitrella patens*, and had been conserved throughout (Supplementary Figure S13). Therefore, the atypical characteristics of DCL1-A that we show in this work, namely its intrinsically disordered nature and folding upon binding the substrate, are most probably essential for the function of the whole protein in miRNA processing.

## ACCESSION NUMBERS

Chemical shift data were deposited in the Biological Magnetic Resonance Data Bank under the BRMB ID 19105 for free DCL1-A and 19104 for folded DCL1-A in complex with dsRNA.

## SUPPLEMENTARY DATA

Supplementary Data are available at NAR Online.

SUPPLEMENTARY DATA
