# Supplementary material for: Induced folding in RNA recognition by Arabidopsis thaliana DCL1
Source: Nucleic Acids Res. 2015 Jun 22;43(13):6607–19. doi: 10.1093/nar/gkv627 (PMC4513881; doi:10.1093/nar/gkv627)
Supplement: SUPPLEMENTARY DATA [file supp_43_13_6607__index.html]

Induced folding in RNA recognition by Arabidopsis thaliana DCL1 — Induced folding in RNA recognition by Arabidopsis thaliana DCL1 — SUPPLEMENTARY DATA 

# Induced folding in RNA recognition by *Arabidopsis thaliana* DCL1

## SUPPLEMENTARY DATA

- SUPPLEMENTARY DATA
