# Supplementary material for: Induced folding in RNA recognition by Arabidopsis thaliana DCL1
Source: Nucleic Acids Res. 2015 Jun 22;43(13):6607–19. doi: 10.1093/nar/gkv627 (PMC4513881; doi:10.1093/nar/gkv627)
Supplement: SUPPLEMENTARY DATA [file supp_gkv627_nar-01080-r-2015-File010.pdf]

Supplementary Information

Induced folding in RNA recognition by *Arabidopsis thaliana* DCL1

Irina P. Suarez[a,b], Paula Burdisso[a,b], Matthieu P.M.H. Benoit[c,d,e,1], Jèrôme Boisbouvier[c,d,e] and Rodolfo M. Rasia[a,b]*.

Description of the two step equilibrium binding model

$a + b$ <=> c <=> d

Where:

a = free RNA

b = Free unfolded protein

c = complex with partially folded protein

d = complex with folded protein

Equilibrium constants:

$K=c/ab$; first equilibrium constant

$J=d/c$; second equilibrium constant

Concentrations:

$A=a+c+d$; total RNA

$B=b+c+d$; total protein

$$a = A - c - d$$

$$b = B - c - d$$

$$ab - \frac{c}{K} = 0$$

$$(A - c - d)(B - c - d) - \frac{c}{K} = 0$$

In the NMR titration experiments, we go through conditions where A≈B >> 1/K. Concentrations of all components can be calculated from the values A, B, K and J through quadratic equations.

Solve for *c* (complex with partially folded protein)

$$d = Jc$$

$$(A - c - Jc)(B - c - Jc) - \frac{c}{K} = 0$$

$$\frac{AB}{(1+J)^2} - c\left[\frac{A+B}{(1+J)} - \frac{1}{K(1+J)^2}\right] + c^2 = 0$$

Solve for *d* (complex with partially folded protein)

$$c = d/J$$

$$(A - d/J - d)(B - d/J - d) - \frac{d}{JK} = 0$$

$$\frac{AB}{(1+1/J)^2} - d\left[\frac{A+B}{(1+1/J)} - \frac{1}{JK(1+1/J)^2}\right] + d^2 = 0$$

Finally, *a* and *b* can be obtained as

$$a = A - c - d$$

$$b = B - c - d$$

In the NMR titration, we know the values of A and B, we fix the value of J as 5, based on the intensity ratio of the folded and unfolded signals in excess of RNA, and we obtain the evolution of *c* from the evolution of the chemical shifts of the unfolded forms in fast exchange during the titration. We obtain an estimate for *K* through non-linear fitting of the data points in Figure 7 to the solution of the quadratic equation for *c*.

The fluorescence anisotropy assays are carried out with $B<K<<A$. In this conditions, $a \approx A$. We measure the ratio between bound RNA (high anisotropy) and free RNA (low anisotropy), that is $(c+d)/B$. Considering the dissociation constants $K_D = ab/c$ and $J_D = c/d$, we get:

$$B = b + \frac{Ab}{K_D} + \frac{Ab}{K_D J_D}$$

From which we get to:

$$\frac{c+d}{B} = \frac{A}{\frac{K_D J_D}{J_D + 1} + A}$$

That is, we obtain an apparent dissociation constant $Kapp = K_D J_D/(J_D+1)$. Using the values obtained from the NMR titration (K_D=2µM and J_D=0.2), the expected value for *Kapp* is 0.33 µM, in good agreement with the measured value (300±50 nM).

Primers used for site directed mutagenesis

Y1751F:	fwd	5'-GCAGAAGGGTTAGAATTCAAAGCGAGTAGGAGTGG-3'
	rev	5'-CCACTCCTACTCGCTTTGAATTCTAACCCTTCTGC-3'
Q1783K:	fwd	5'-GCAGAAGAAAATGGCTAAAAAGCTAGCTGCGAGGAACG-3'
	rev	5'-CGTTCCTCGCAGCTAGCTTTTTAGCCATTTTCTTCTGC-3'
E1747W:	fwd	5'-CGGTGCCAGCAACAAGCATGGGGGTTAGAATACAAAGCG-3'
	rev	5'-CGCTTTGTATTCTAACCCCCATGCTTGTTGCTGGCACCG-3'
Q1778D:	fwd	5'-GGAGTAGCGCAAAACCCGGACAAGAAAATGGCTCAAAAGC-3'
	rev	5'-GCTTTTGAGCCATTTTCTTGTCCGGGTTTTGCGCTACTCC-3'
KK1779-80AA:	fwd	5'-GGAGTAGCGCAAAACCCGCAGGCGGCAATGGCTCAAAAGCTAGCTGC-3'
	rev	5'-GCAGCTAGCTTTTGAGCCATTGCCGCCTGCGGGTTTTGCGCTACTCC-3'

Supplementary Figure 1

MetaPrDOS (Ishida & Kinoshita, 2008) (http://prdos.hgc.jp/cgi-bin/meta/top.cgi) disorder prediction for DCL1 dsRBDs region.

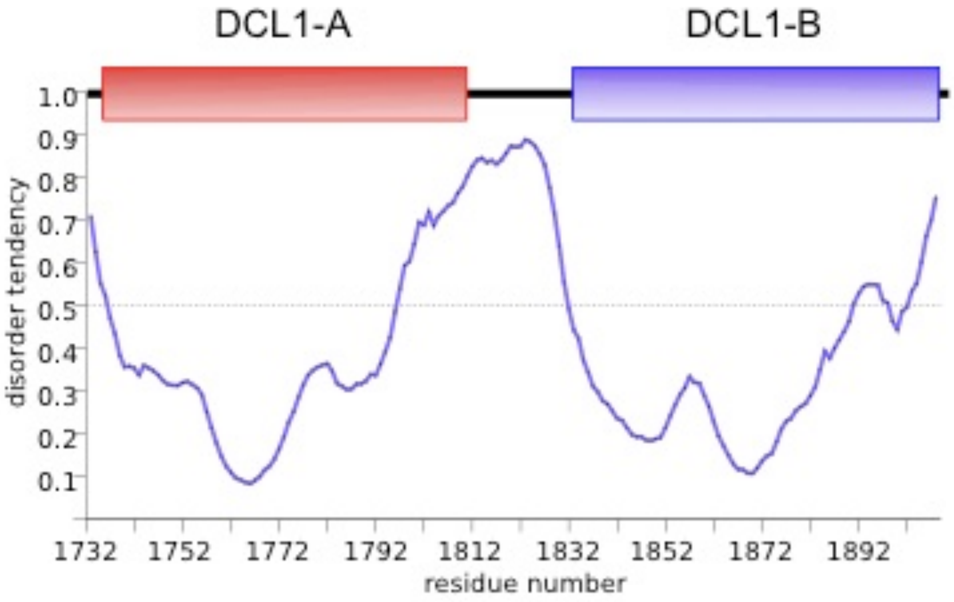

Supplementary Figure 2

Spectra of the protein constructs DCL1-A, DCL1-AB, DCL1-A/N. Left, overlay of the spectra corresponding to DCL1-A (black) and DCL1-A/N (green). Right, overlay of the spectra corresponding to DLC1-A (black), DCL1-B (blue) and DCL1-AB (red). The sequences of each construct and the domain definition are schematized below.

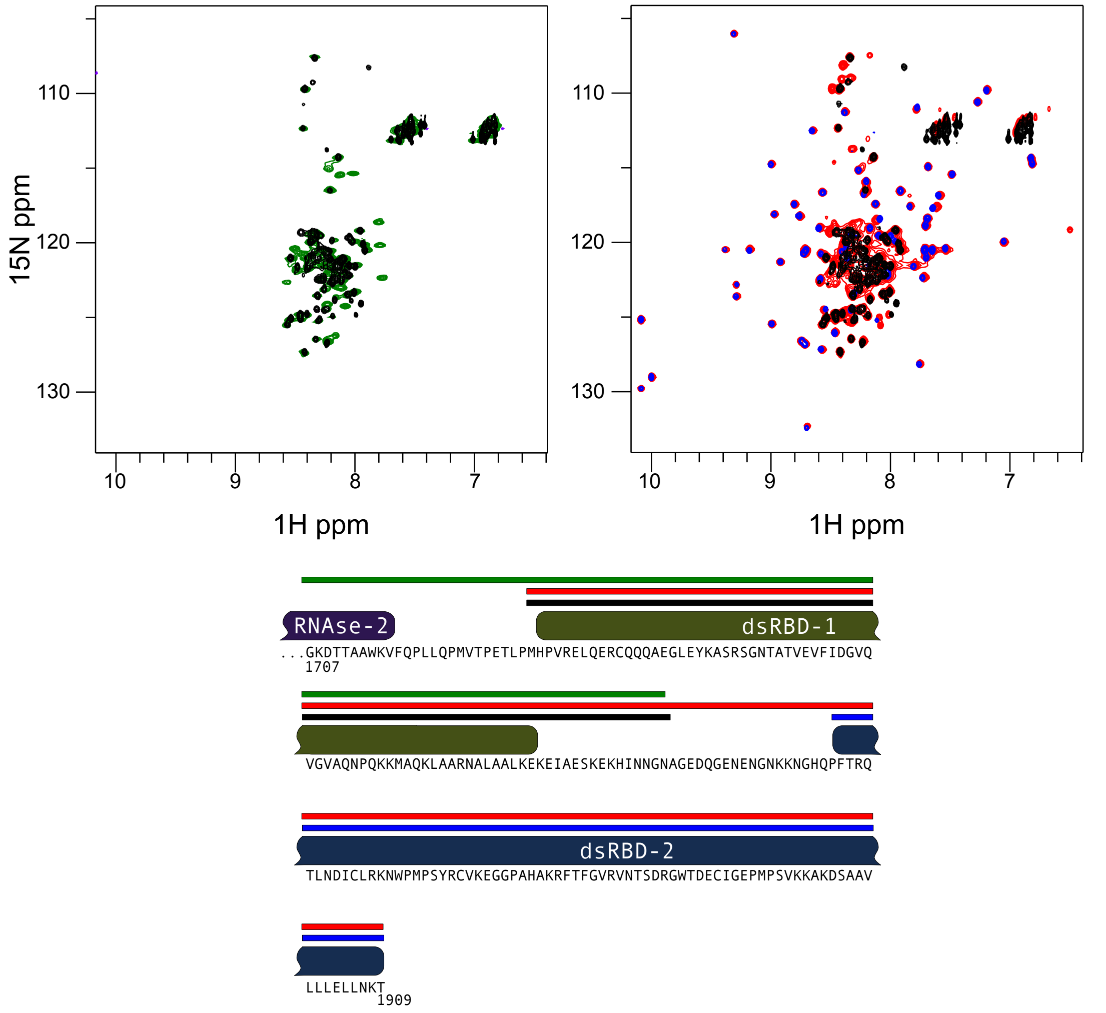

Supplementary Figure 3

A. ^{1}H-^{15}N HMQC spectra of DCL1-A in buffer (green) and in 8M urea (blue) at 298K. B. ^{1}H-^{15}N HMQC spectra of DCL1-A in buffer at 298K (pink) and at 278K (dark blue).

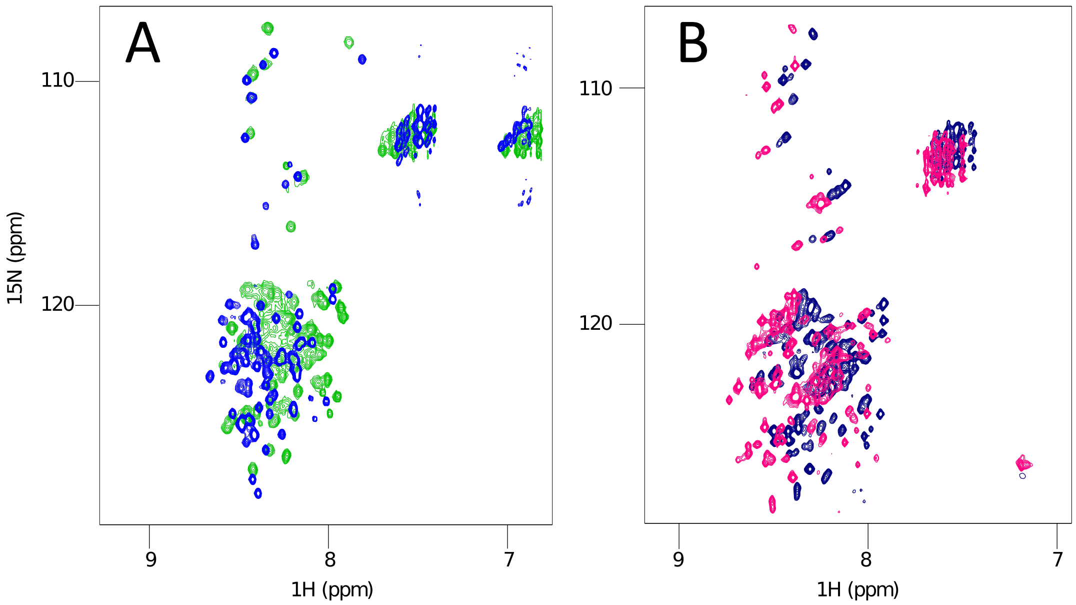

Supplementary Figure 4

A. Alignment of DCL1-A with dsRBDs of known structure. The secondary structure elements are shown on top. Note the deletion in loop beta1-beta2 in all three dicer dsRBDs (highlighted with a red rectangle). The alignment was calculated using Muscle (Edgar, 2004). B. Sequence distance tree corresponding to the alignment. C. Sequence alignment of DCL1-A with its closest homologs, mouse Dicer and *K. polysporus* Dicer.

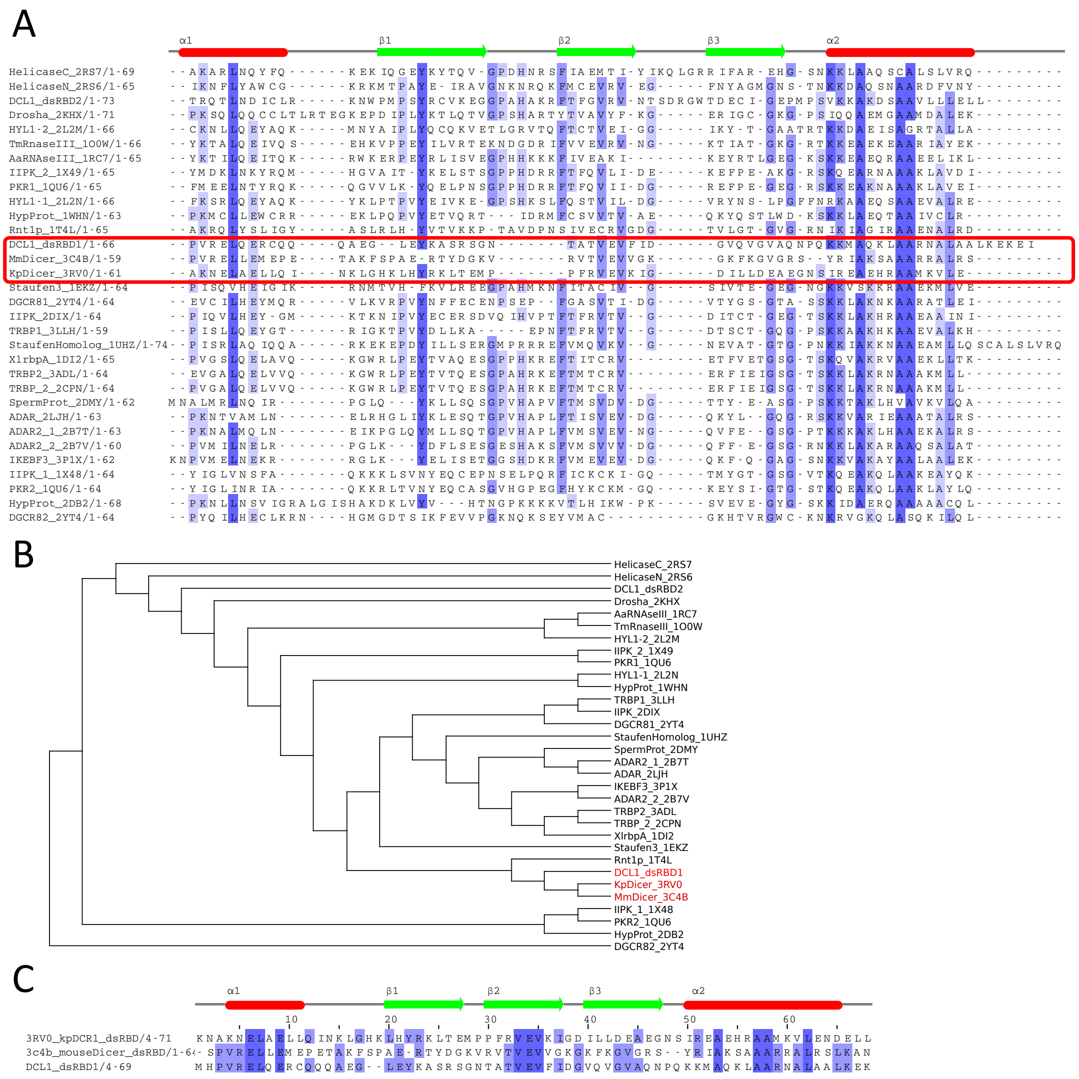

Supplementary Figure 5

The 1H-1D NMR spectrum Dicer dsRBDs. In blue, the folded *Mus musculus* Dicer dsRBD. In red, the intrinsically disordered DCL1-A.

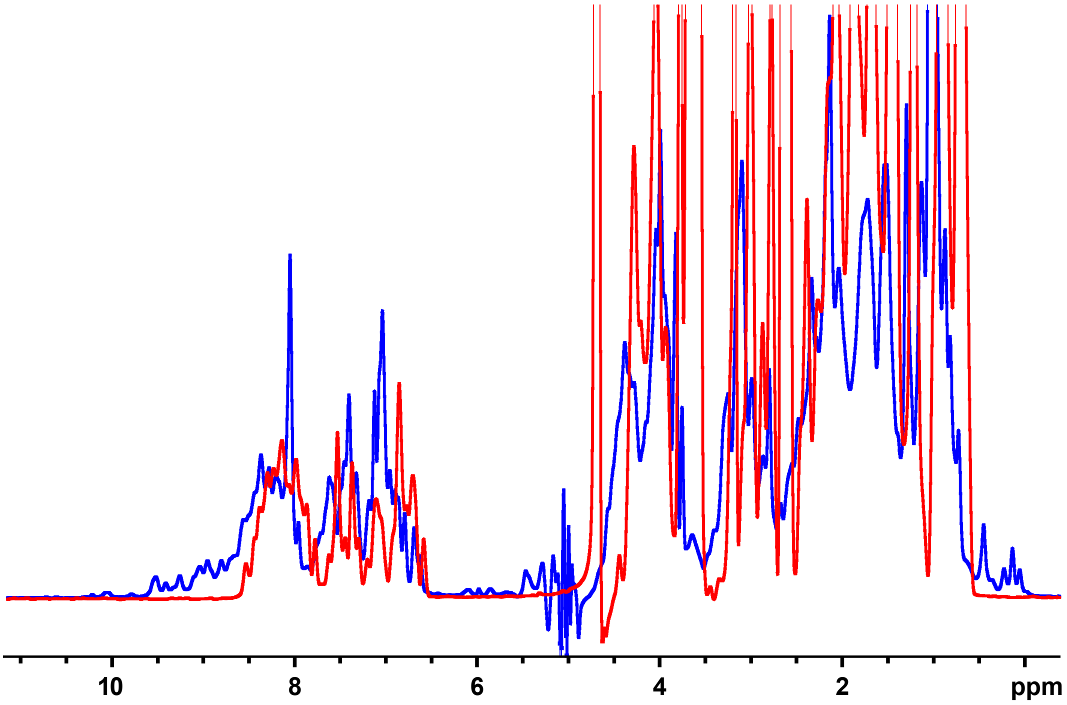

Supplementary Figure 6

Far UV CD spectra of HYL1-dsRBD1 (Burdisso *et al.*, 2014) and DCL1-A. The spectrum from DCL1-A corresponds to the component of the titration obtained by multivariate curve resolution–alternating least-squares.

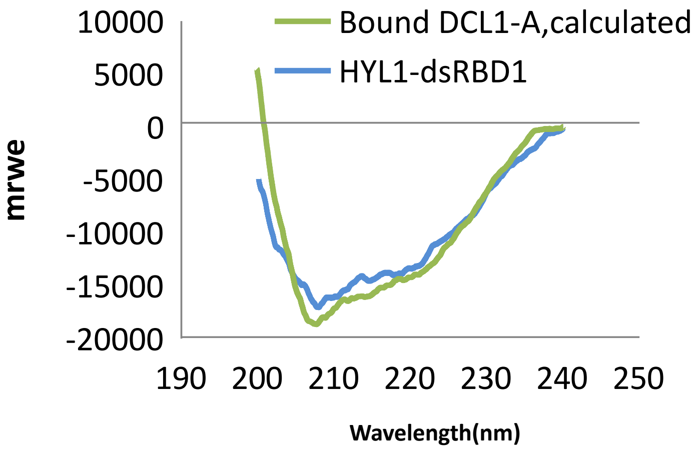

Supplementary Figure 7

A. Binding of DCL1-A to full-length pri-miR172a followed by CD. Spectra of the titration are gradient colored from purple (free RNA) to light blue (RNA + 10 equivalents protein). Spectra correspond to 1 µM pri-miR172a with the addition of 0, 0.2, 0.5, 0.7, 0.8, 0.9, 1.0, 1.1, 1.2, 1.3, 1.5, 2.0, 2.5, 3.0 and 10.0 equivalents of DCL1-A. B. Spectral components obtained from MCD-ALS analysis of the titration experiment: Light blue, bound protein; dark blue, free RNA; green, RNA complex 1; red, RNA complex 2. C. Evolution of the concentration of the four species detected during the titration, color-coded as in the center panel. The point at 10 equivalents was omitted for clarity, but gives a value of ca. 10 for the bound protein component. D. Ionic strength dependence of DCL1-A RNA binding. Dissociation of the complex induced by increasing the NaCl concentration from 5 to 175 mM was followed by CD. E. Comparison of ionic strength dependence of RNA binding by DCL1-A and DCL1-B. Dissociation of DCL1-B:dsRNA complex was followed by Fluorescence Anisotropy (Burdisso *et al.*, 2012), whereas the DCL1-A data correspond to the curves shown in panel D. F. DCL1-A dissociates from dsDNA at increasing ionic strength.

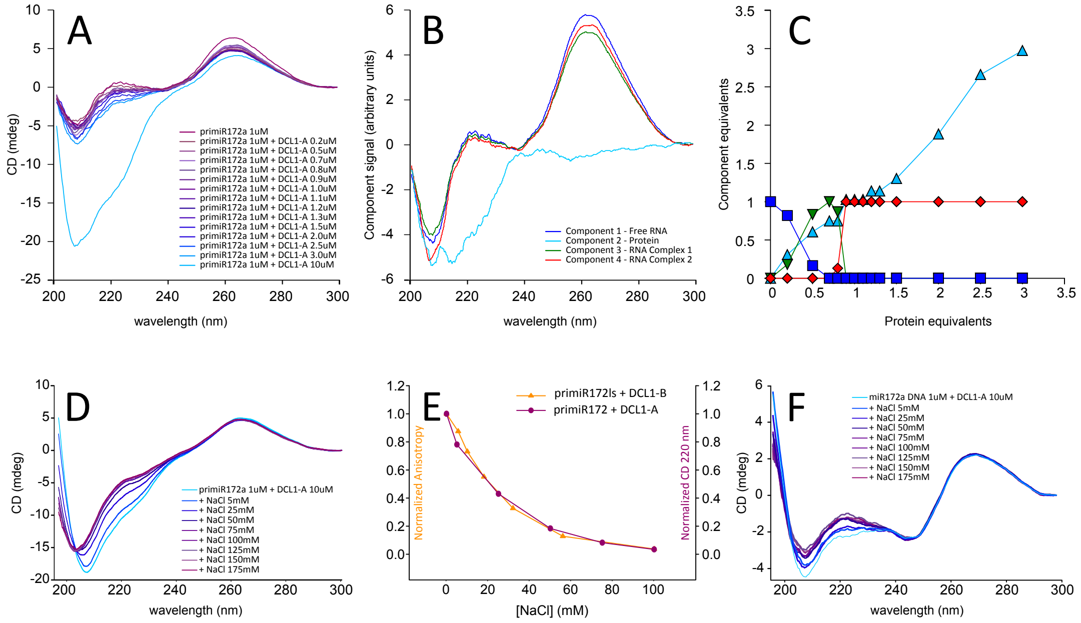

Supplementary Figure 8

ZZ exchange spectra of the DCL1-A:pri-miR172-ls complex. The spectra were acquired with no mixing time (red) and 0.2s mixing time (green). Selected exchange peaks and the corresponding non-exchanged signals are highlighted with their rectangular patterns.

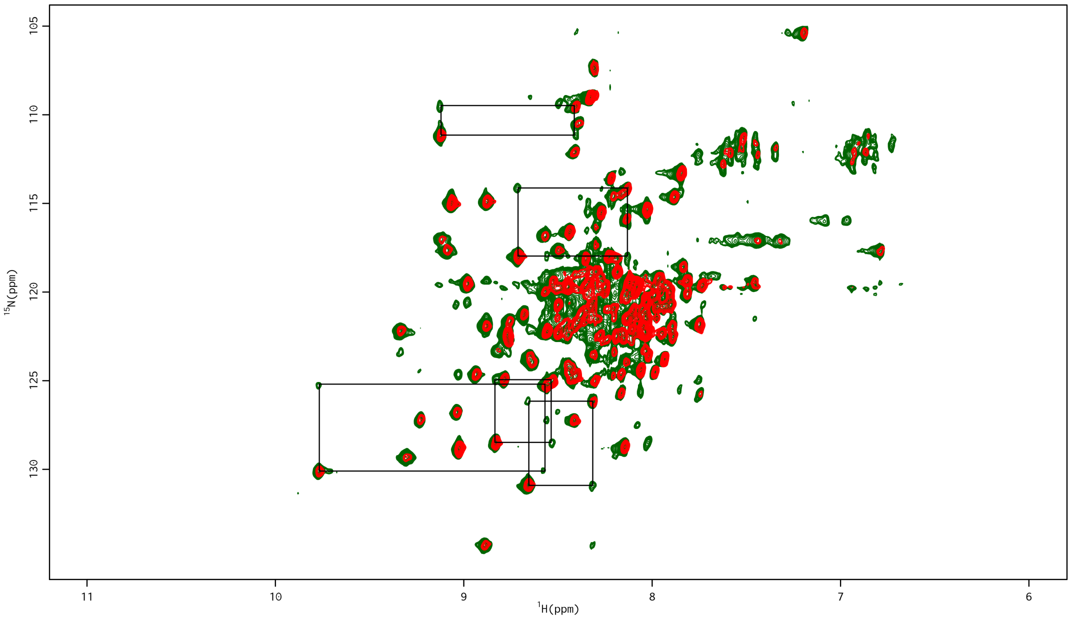

Supplementary Figure 9

Folding of DCL1-A requires dsRNA as partner. A, B. Addition of up to 0.56 M NaCl (orange spectra) induces only minor changes in the 1H-15N HSQC spectrum of free DCL1-A (blue spectra). The spectrum of the folded form (pink) is shown for comparison. C. Chemical shift changes of the unfolded-bound form of DCL1-A are not correlated with those observed at high ionic strength. D. The secondary structure of free DCL1-A is not dependent on the presence of NaCl, as shown by the equivalence of the far UV CD spectra. E. dsDNA binding induces minor changes in the secondary structure of DCL1-A (green vs. blue spectra), but the change in conformational sampling is negligible compared to that seen in the dsRNA complex (pink).

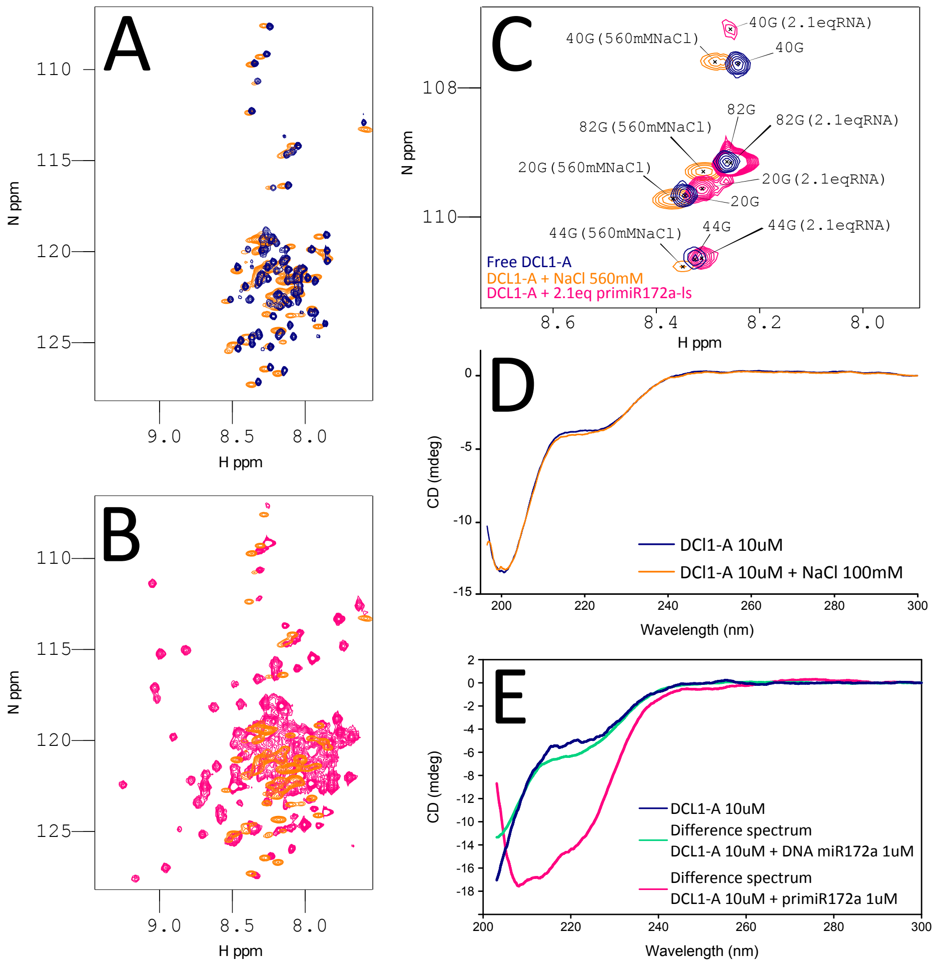

Supplementary Figure 10

Imino region of the 1H NMR spectra acquired during the titration of DCL1-A with pri-miR172-ls RNA construct. From bottom to top, spectra at 0.25, 0.51. 0.76. 1.01 and 1.52 equivalents RNA. Sharper signals at 1.52 equivalents indicate the presence of free RNA in the sample. B. Assignment of the imino region of pri-miR172-ls. Green lines show the connectivities corresponding to the loop helix (bases 8-16) and red lines those corresponding to the lower stem helix (bases 3-6). C. 1H-15N HSQC spectrum of the construct.

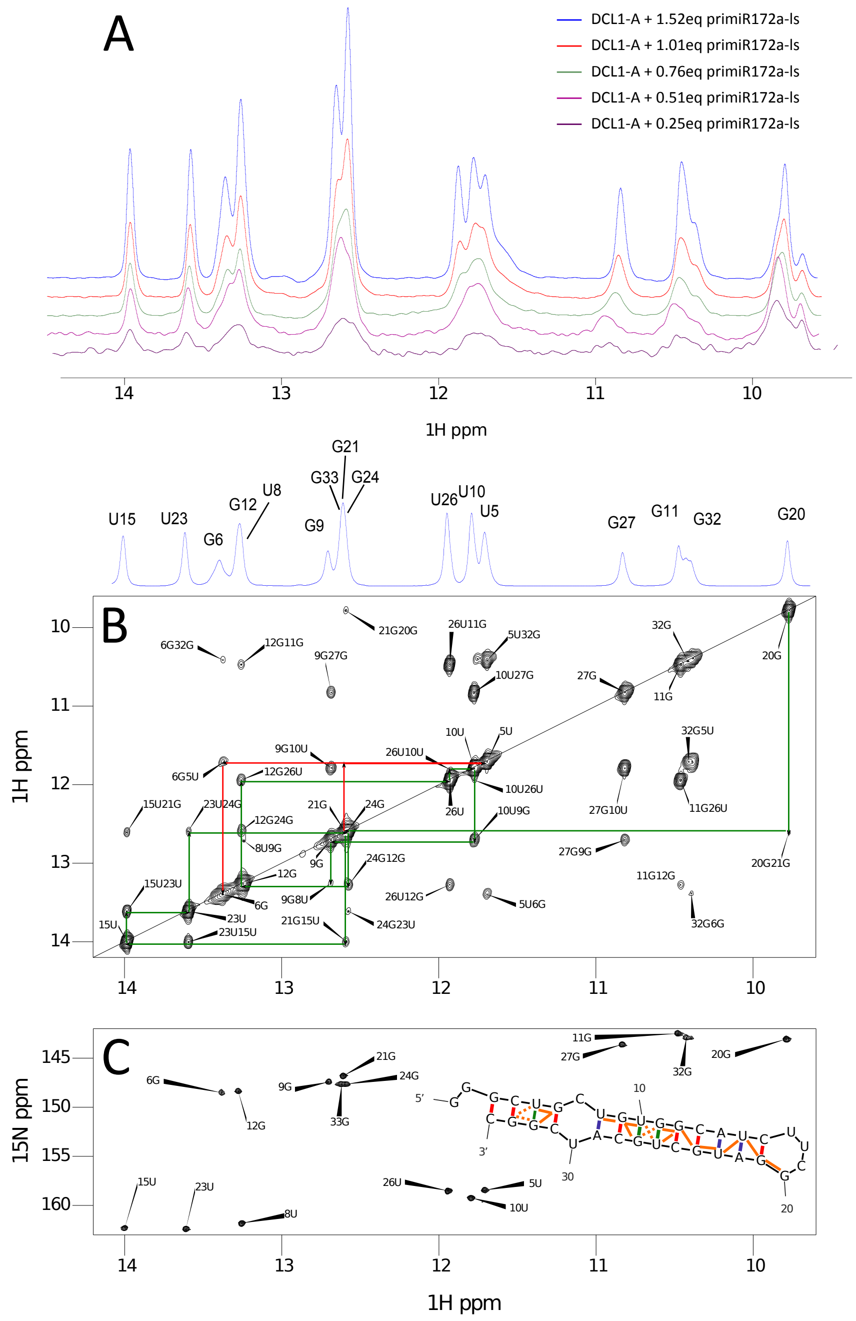

Supplementary Figure 11

Analysis of the secondary chemical shifts of C_{alpha} and C' of the bound structured form (top), the bound unstructured form (middle) and the free unstructured form (bottom) of DCL1-A.

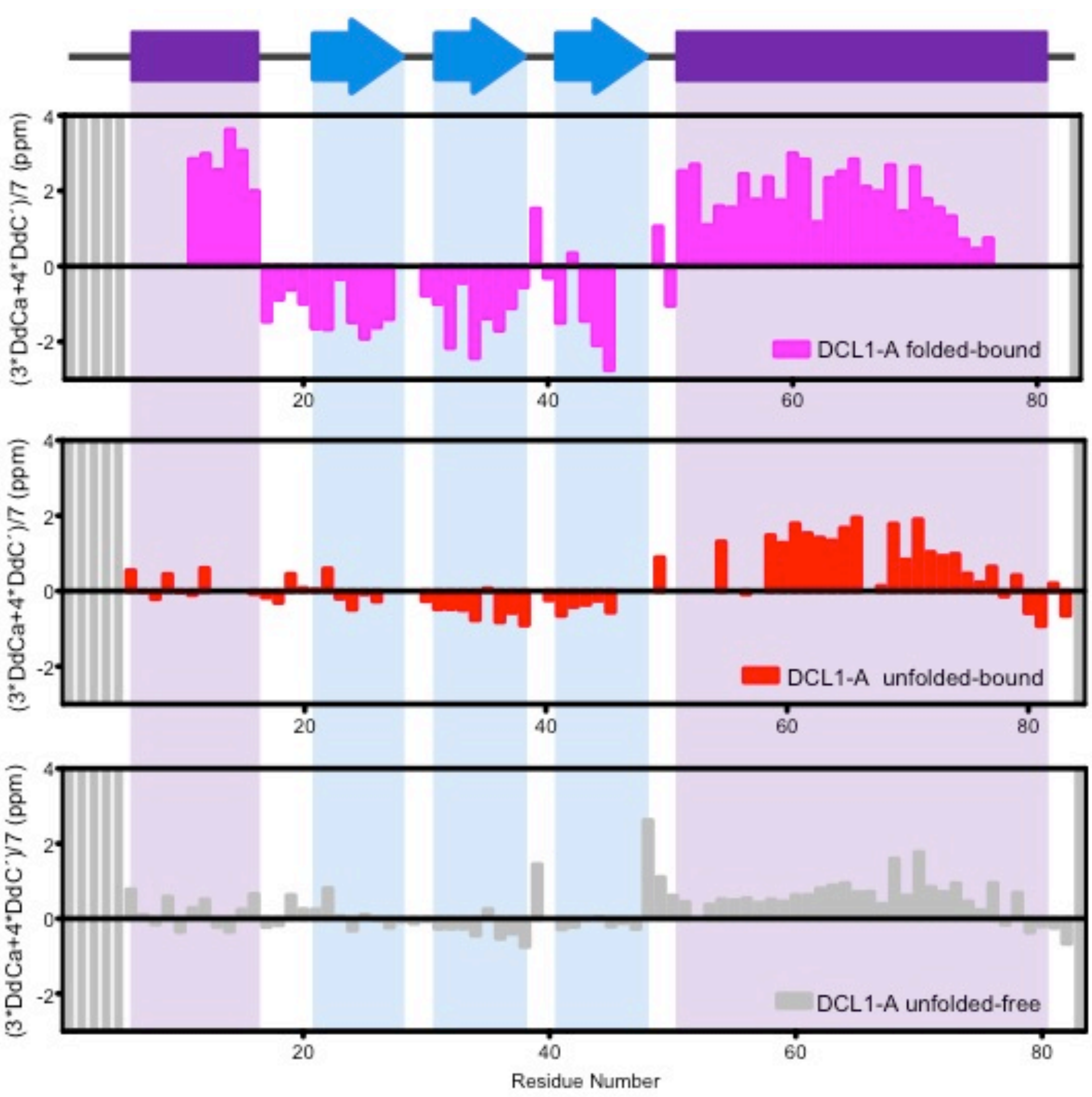

Supplementary Figure 12

A. Structure of the folded bound form of DCL1-A showing the residues mutated (left) and the result of the mutations (right). B. Amide region of the 1H 1D NMR spectra of the mutant proteins designed to stabilize the folded form of DCL1-A. The spectrum of mouse Dicer dsRBD is shown on top for comparison.

A.

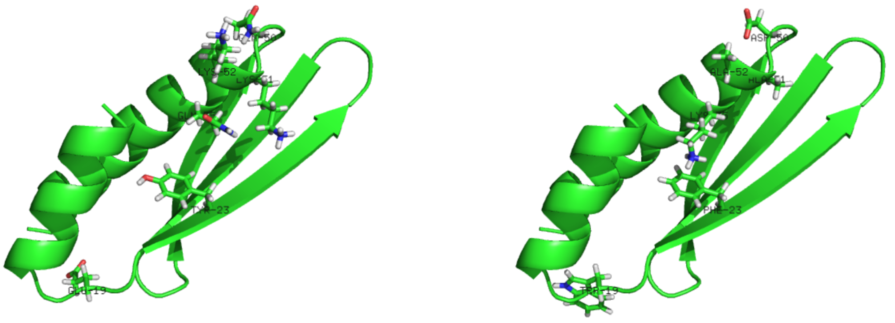

B.

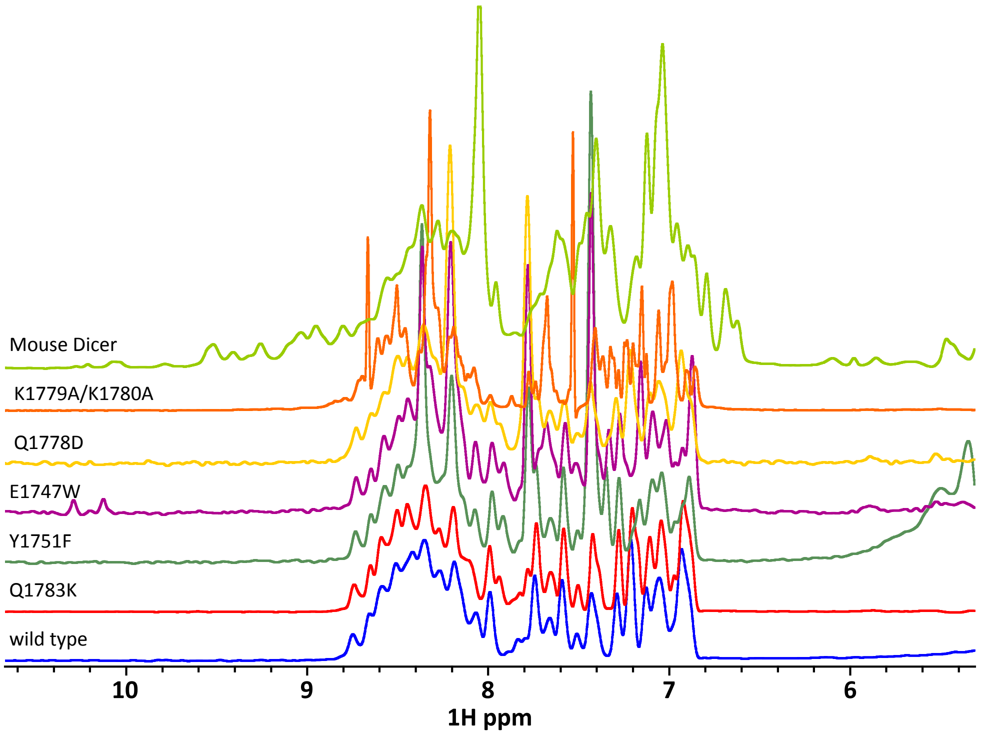

Supplementary Figure 13

Cladogram and alignments showing the sequence similarities between dsRBD-A domains in DCL proteins from different plants. The sequences corresponding to the first domain of the sequenced plant DCLs were aligned using MUSCLE (Edgar, 2004), and the cladogram was constructed based on the alignments using FastTree (Price *et al.*, 2010). Residues on the alignments are colored in blue shade according to conservation.

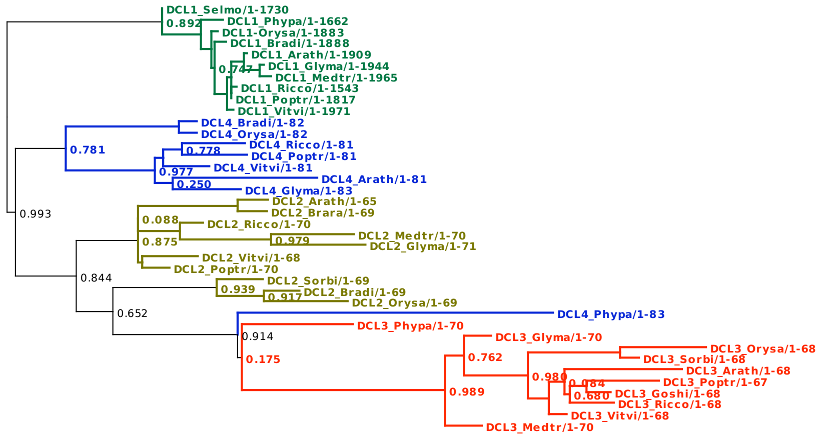

DCL1-A

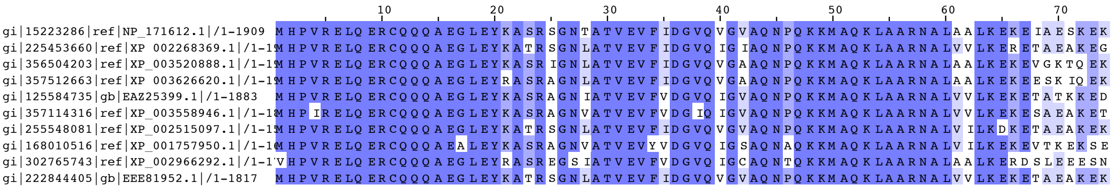

DCL2-A

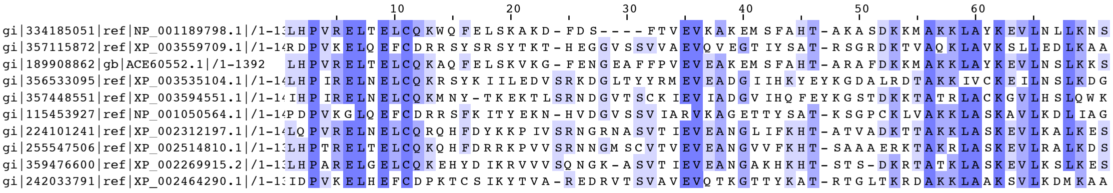

DCL3-A

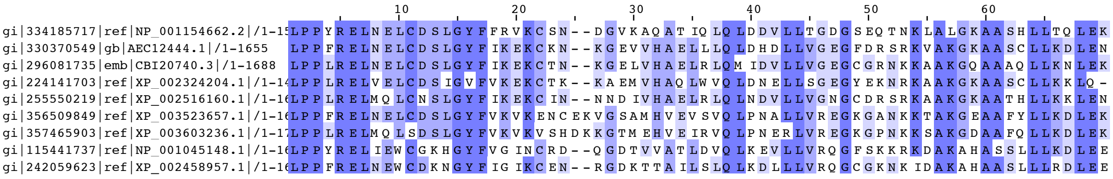

DCL4-A

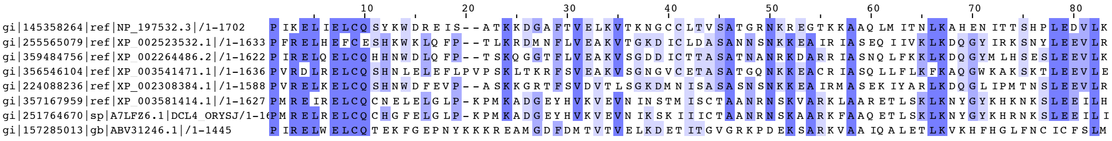

References

1. Ishida, T. & Kinoshita, K. (2008). Prediction of disordered regions in proteins based on the meta approach. *Bioinformatics* **24**, 1344–1348.

2. Burdisso, P., Suarez, I. P., Bologna, N. G., Palatnik, J. F., Bersch, B., and Rasia, R. M. (2012) Second double-stranded RNA binding domain of dicer-like ribonuclease 1: structural and biochemical characterization. *Biochemistry* **51**, 10159–66

3. Burdisso, P., Milia, F., Schapire, A. L., Bologna, N. G., Palatnik, J. F., and Rasia, R. M. (2014) Structural Determinants of Arabidopsis thaliana Hyponastic Leaves 1 Function In Vivo. *PLoS One*. **9**, e113243

4. Edgar, R.C. (2004). MUSCLE: multiple sequence alignment with high accuracy and high throughput. *Nucleic Acids Research* **32**, 1792–1797.

5. Price, M.N., Dehal, P.S. & Arkin, A.P. (2010). FastTree 2 – Approximately Maximum-Likelihood Trees for Large Alignments. *PLoS ONE* **5**, e9490.
